# Synthesis of an α-phosphono-α,α-difluoroacetamide analogue of the diphosphoinositol pentakisphosphate 5-InsP_7_†
†Electronic supplementary information (ESI) available: Data deposition: atomic coordinates and structure factors have been deposited in the Protein Data Bank (PDB ID code 6N5C). See DOI: 10.1039/c9md00163h


**DOI:** 10.1039/c9md00163h

**Published:** 2019-06-07

**Authors:** Andrew M. Riley, Huanchen Wang, Stephen B. Shears, Barry V. L. Potter

**Affiliations:** a Medicinal Chemistry and Drug Discovery , Department of Pharmacology , University of Oxford , Mansfield Road , Oxford OX1 3QT , UK . Email: barry.potter@pharm.ox.ac.uk ; Fax: +44 (0)1865 271853 ; Tel: +44 (0)1865 271945; b Inositol Signaling Group , Laboratory of Signal Transduction , National Institute of Environmental Health Sciences , National Institutes of Health , Research Triangle Park , North Carolina , USA

## Abstract

Diphosphoinositol phosphates (PP-InsPs) are an evolutionarily ancient group of signalling molecules that are essential to cellular and organismal homeostasis. As the detailed mechanisms of PP-InsP signalling begin to emerge, synthetic analogues of PP-InsPs containing stabilised mimics of the labile diphosphate group can provide valuable investigational tools. We synthesised 5-PCF_2_Am-InsP_5_ (**1**), a novel fluorinated phosphonate analogue of 5-PP-InsP_5_, and obtained an X-ray crystal structure of **1** in complex with diphosphoinositol pentakisphosphate kinase 2 (PPIP5K2). 5-PCF_2_Am-InsP_5_ binds to the kinase domain of PPIP5K2 in a similar orientation to that of the natural substrate 5-PP-InsP_5_ and the PCF_2_Am structure can mimic many aspects of the diphosphate group in 5-PP-InsP_5_. We propose that **1**, the structural and electronic properties of which are in some ways complementary to those of existing phosphonoacetate and methylenebisphosphonate analogues of 5-PP-InsP_5_, may be a useful addition to the expanding array of chemical tools for the investigation of signalling by PP-InsPs. The PCF_2_Am group may also deserve attention for wider application as a diphosphate mimic.

## Abstract 2

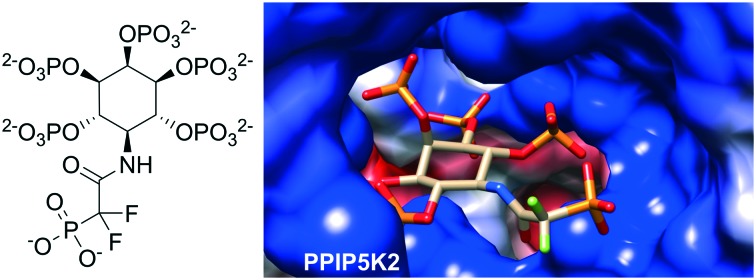
A synthetic, fluorinated analogue of 5-InsP_7_ binds to the kinase domain of PPIP5K2, suggesting new strategies for designing diphosphoinositol phosphate mimics.

## Introduction

**Fig. 1:**
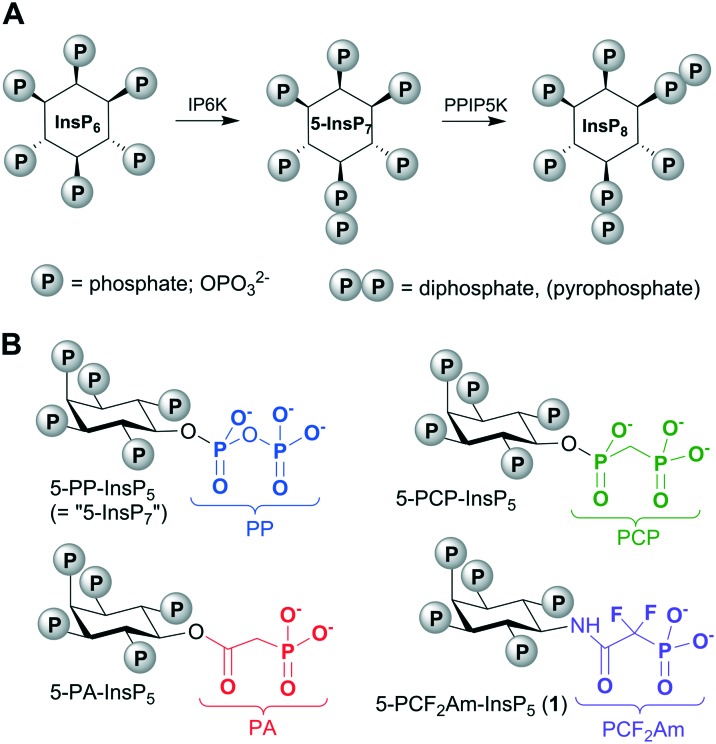
A. Inositol hexakisphosphate (InsP_6_) is phosphorylated on P-5 by IP6K to give 5-PP-InsP_5_, also known as “5-InsP_7_”. Further phosphorylation at P-1 by PPIP5K gives 1,5-[PP]_2_-InsP_4_, (“InsP_8_”). B. Stabilised analogues of 5-PP-InsPP_5_ containing methylenebisphosphonate (PCP), phosphonoacetate (PA) and phosphonodifluoroacetamide (PCF_2_Am) mimics of the natural PP group. IP6K, inositol hexakisphosphate kinase; PPIP5K, diphosphoinositol pentakisphosphate kinase.

The diphosphoinositol phosphates (PP-InsPs, “inositol pyrophosphates”) are of fundamental importance to all eukaryotes, with pivotal roles in cellular and organismic metabolic homeostasis (Fig. 1).^1^ The acute clustering of monophosphate and diphosphate groups around the hexahydroxycyclohexane ring of *myo*-inositol (Ins) endows the PP-InsPs with the most concentrated three-dimensional array of phosphate groups found in Nature.^1^ The PP-InsPs are formed through the enzymatic phosphorylation of *myo*-inositol hexakisphosphate (InsP_6_, Fig. 1) by inositol hexakisphosphate kinases (IP6Ks) and diphosphoinositol pentakisphosphate kinases (PPIP5Ks).^2^ Among the PP-InsPs, 5-diphospho-*myo*-inositol pentakisphosphate (5-PP-InsP_5_, also known as “5-InsP_7_”) is both the most abundant and the most intensively studied member of this signalling family.

An understanding of the molecular actions of PP-InsPs by their addition to cell-free systems can be confounded by the enzymatic and chemical instability of the diphosphate (PP) component. Therefore, we and other workers have developed more stable, synthetic versions of PP-InsPs in which the PP groups are replaced with phosphonoacetate (PA)^3^,^4^ or methylenebisphosphonate (PCP)^4^–^7^ groups (Fig. 1).^8^ Thus, a tethered version of 5-PCP-InsP_5_ was recently used to search for novel binding proteins for PP-InsPs.^9^ Metabolically-stable PP-InsP analogues can also be informative for structural analysis of enzyme/substrate crystal complexes. For example, we have used 5-PA-InsP_5_ to reveal a previously unidentified ligand capture site on PPIP5K2.^10^

While PA analogues are relatively easy to synthesise, the carboxylic ester of the PA group could be prone to chemical hydrolysis at high pH and/or enzymatic cleavage by cellular esterases.^11^ In both PA and PCP analogues, the bridging oxygen of the diphosphate group is replaced by a methylene (CH_2_) group, making the resulting carbon–phosphorus bonds resistant to hydrolysis in comparison to the native oxygen–phosphorus bonds.^12^ However, the CH_2_ group in methylene phosphonates is less electronegative than the bridging oxygen atom of phosphates, causing an increase in the p*K*_a_ value of the methylene phosphonic acid in its second deprotonation. This can mean that a methylene phosphonate analogue is less strongly ionised than the phosphate equivalent at physiological pH, potentially leading to a decreased affinity of the analogue for protein binding sites.^12^ A well-established approach to this problem, originally developed by Blackburn and co-workers,^13^ involves replacing the phosphonate CH_2_ group with difluoromethylene (CF_2_). The more electronegative CF_2_ group increases the acidity of the phosphonic acid and, in addition, the CF_2_ group itself has greater electronic and steric similarity to the bridging oxygen atom of a phosphate than does CH_2_.^14^ The difluoromethylenephosphonate (PCF_2_) group has been particularly useful as a phosphate mimic in the development of protein tyrosine phosphatase inhibitors.^15^,^16^

In nucleotide chemistry, the difluoromethylene-bisphosphonate (PCF_2_P) motif has been used to mimic PP in stable analogues of nucleoside diphosphates and triphosphates.^17^ Although the PCF_2_P group has been proposed as a potential PP mimic in stabilised analogues of PP-InsPs, this was anticipated to present a considerable synthetic challenge,^5^,^18^ and while several PCP-InsPs have been synthesised,^4^–^7^ no fluorinated equivalents have yet been disclosed.

In the current report, we explore an alternative approach to a fluorinated isopolar analogue of a PP-InsP, building on our earlier work with phosphonoacetic acid (PA) esters. Although replacing the CH_2_ group in the β-ketophosphonate fragment of a PA ester with CF_2_ is possible, the resulting difluoroacetate ester would be very labile to hydrolysis. Therefore, we chose to replace the ester of PA with a stable amide linkage, allowing the inclusion of the electron-withdrawing CF_2_ group in place of CH_2_. In the resulting analogue, 5-PCF_2_Am-InsP_5_ (**1**, Fig. 1), the terminal phosphonate group should more closely resemble the corresponding β-phosphate group of 5-PP-InsP_5_ in its electronic properties than in either the PA or the PCP equivalents. The α-phosphono-α,α-difluoroacetamide (PCF_2_Am) unit lacks a close equivalent to the α-phosphate of PP, but the amide carbonyl retains the potential to accept H-bonds and the rigidity of the amide structure itself may confer advantages at some binding sites. To the best of our knowledge, the PCF_2_Am motif^19^ has not previously been explored as a diphosphate isostere, although it has been used successfully in analogues of 1,3-bisphosphoglyceric acid as inhibitors of phosphoglycerate kinase^20^ and in the design of inhibitors of aspartate carbamoyltransferase^21^ and protein tyrosine phosphatases.^16^

## Results and discussion

### Synthesis of 5-PCF_2_-Am-InsP_5_ (**1**)

**Scheme 1:**
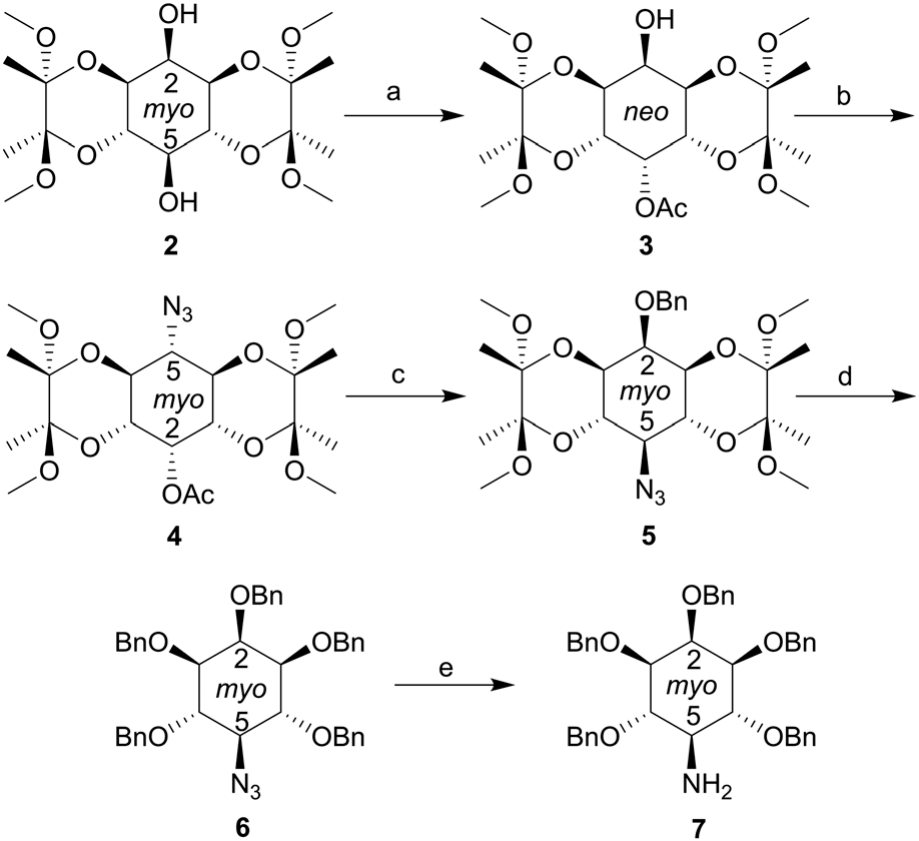
Synthesis of 5-deoxy-5-amino-inositol **7**. Reagents and conditions: a. see ref. 22; b. i. Tf_2_O, CH_2_Cl_2_, pyridine, –78 °C to r.t.; ii. NaN_3_, DMF, r.t., 86%; c. i. LiOH·H_2_O, MeOH, THF, H_2_O; ii. NaH, BnBr, DMF, 93%; d. i. CH_2_Cl_2_, 95% aqueous TFA; ii. NaH, BnBr, DMF, 91%; e. LiAlH_4_, THF, 100%. Bn, benzyl.

To construct compound **1**, we needed to synthesise an appropriately protected 5-deoxy-5-amino-*myo*-inositol intermediate (Scheme 1). We have previously shown^22^ that regioselective sulfonylation of butanediacetal (BDA) protected *myo*-inositol **2** (ref. 3, 22 and 23) using triflic anhydride, followed by solvolysis using wet dimethylacetamide, gives inversion of configuration at C-5, to produce the *neo*-inositol acetate derivative **3***via* an iminium ion intermediate.^22^ In the present work, we used a second triflate esterification of the free OH group in **3** followed by treatment with sodium azide in DMF to give a second configurational inversion, returning us to the *myo*-inositol configuration in the 5-deoxy-5-azido-*myo*-inositol derivative **4**.‡
‡A recent study^26^ has systematically investigated the solvolysis and azidolysis of triflates derived from diol **2**.


**Scheme 2:**
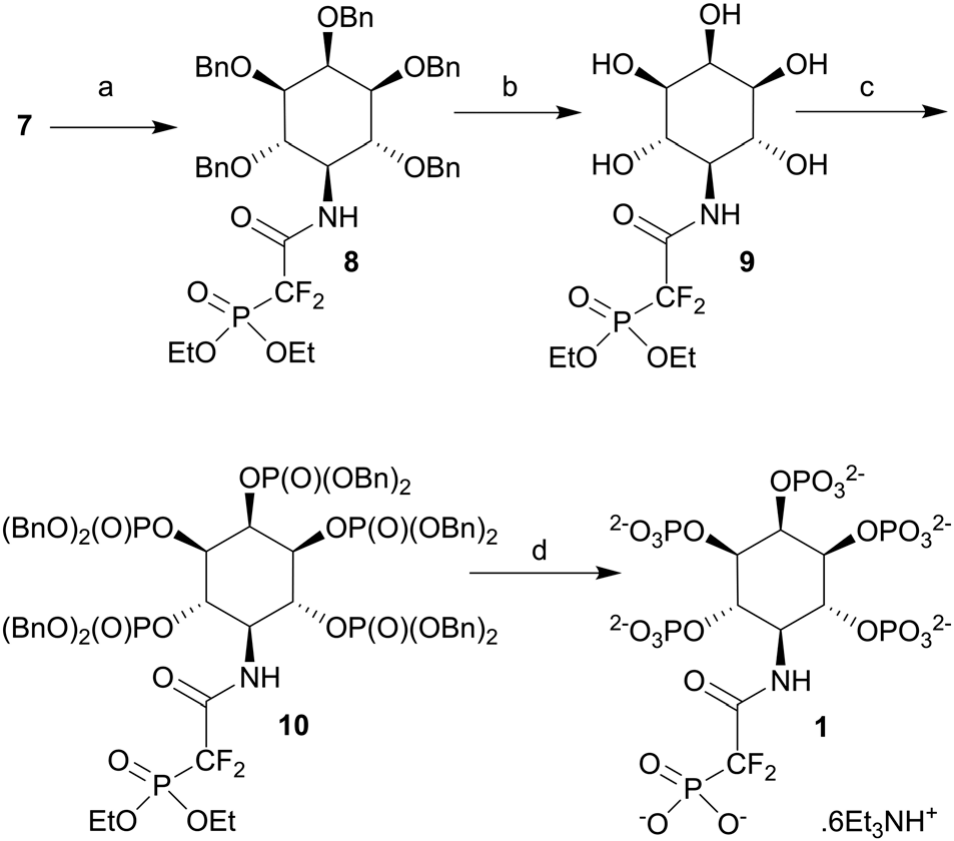
a. (EtO)_2_P(O)CF_2_COOH, EDAC, CH_2_Cl_2_, 87%; b. H_2_, 50 p.s.i., Pd(OH)_2_/C, MeOH, THF, AcOH, 100%; c. i. (BnO)_2_PN^i^Pr_2_, 5-phenyl-1*H*-tetrazole, CH_2_Cl_2_; ii. *m*CPBA, 66%; d. i. TMSBr, CH_2_Cl_2_; ii. MeOH; iii. aqueous triethylammonium bicarbonate, 85%. Bn, benzyl.

Compound **4**, with its combination of acid- and base-labile protecting groups, is a potentially versatile intermediate in itself. However, to simplify the current synthesis, we sequentially replaced these labile protecting groups with benzyl ethers. Thus, replacement of the 2-*O*-acetate ester with a 2-*O*-benzyl ether gave **5**, a precursor for the synthesis of analogues of 5-PP-InsP_4_. Next, the BDA groups were removed and replaced with benzyl ethers to give pentabenzyl 5-deoxy-5-azido-inositol **6**. Reduction of the azide group in **6** using LiAlH_4_ now gave the 5-deoxy-5-amino-inositol **7** in quantitative yield. The next step was to introduce the phosphonodifluoroacetamide unit (Scheme 2). Thus, reaction of **7** with diethyl phosphono-difluoroacetic acid^24^ gave phosphonodifluoroacetamide **8**. The benzyl protecting groups in **8** were removed by catalytic hydrogenolysis over Pd(OH)_2_/C to give pentaol **9**. Phosphitylation using bisbenzyloxydiisopropylaminophosphine activated with 5-phenyl-1*H*-tetrazole then gave the intermediate pentakisphosphite, which was observed by ^31^P NMR, but not isolated. Oxidation of phosphites with *m*CPBA yielded the fully-protected pentakisphosphate **10**. Finally, the benzyl and ethyl protecting groups in **10** were cleanly removed using TMSBr in dichloromethane followed by MeOH.

Compound **1** was isolated as the triethylammonium salt, containing six TEA^+^ ions per molecule of **1**. The non-fluorinated phosphonoacetamide (PCH_2_Am) equivalent of **1** was also initially synthesised to explore synthetic methods (see ESI† for details). During this synthesis, we observed facile exchange of the CH_2_ protons with deuterium from D_2_O during NMR spectroscopy of the PCH_2_Am-containing pentaol intermediate analogous to compound **9**. The deuterium was retained after the subsequent phosphorylation and deprotection steps. Attempts to exchange deuterium back to hydrogen in the final product were unsuccessful, possibly because ionisation of the methylenephosphonate group disfavours the required enolisation (see ESI† for further details). This may suggest a strategy for developing tritiated versions of the non-fluorinated equivalent of **1** and related analogues of PP-InsPs.

The ^31^P NMR spectrum of **1** in D_2_O showed the signal corresponding to the PCF_2_Am phosphorus atom at *δ* 0.64, shifted approximately 13.5 ppm up-field relative to the equivalent signal in the PCH_2_Am equivalent (ESI† Fig. S1). This reflects the profound effect of the CF_2_ group on the electronic properties of the phosphonate group in **1**. Compound **1** was very stable in aqueous solution; the NMR sample showed no sign of decomposition after >1 year in solution in D_2_O.

### Interaction of 5-PCF_2_-Am-InsP_5_ with PPIP5K2

**Fig. 2:**
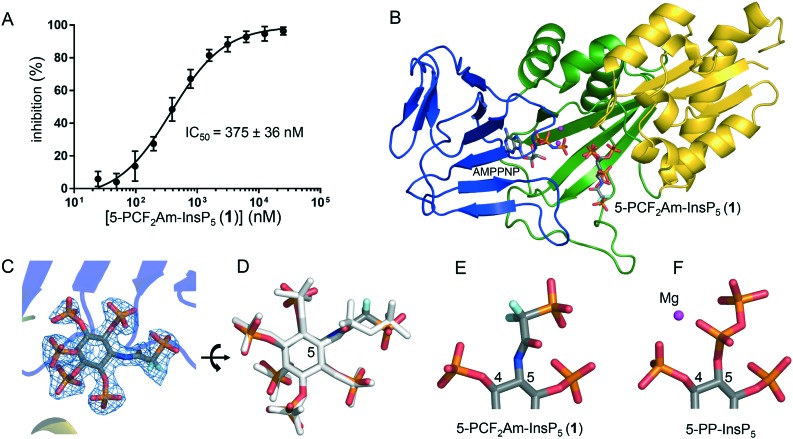
A. Inhibition of human PPIP5K2 by 5-PCF_2_Am-InsP_5_ (**1**) in the presence of 100 nM 1,5-[PP]_2_-InsP_4_. B. Crystallographic analysis of PPIP5K2 in complex with **1** and AMPPNP (PDB code ; 6N5C). C. The 2Fo–Fc electron density map of **1**, contoured at 1.7*σ*, is shown as light blue mesh. PPIP5K2 is shown in cartoon and **1** is shown as a stick model, with carbon atoms coloured dark grey, phosphorus atoms orange, oxygen atoms red, nitrogen atom blue and fluorine atoms cyan. D. Another view of 5-PCF_2_Am-InsP_5_ (**1**) and its overlap with 5-PP-InsP_5_, which is shown as white sticks. Panels E and F show the moieties on position 5 of **1** and the natural substrate 5-PP-InsP_5_ (PDB code, ; 3T9D).

To determine whether **1** could act as a mimic of 5-PP-InsP_5_, we examined its interaction with the kinase domain of human PPIP5K2. We used a “reverse-kinase” assay that records ATP generated from 0.1 mM ADP during the dephosphorylation of 100 nM 1,5-[PP]_2_-InsP_4_. This approach avoids the need to use radiolabelled material and slow-throughput HPLC analysis. Furthermore, the assay of ATP production is inherently more sensitive compared to measuring ATP consumption. Compound **1** inhibited 1,5-[PP]_2_-InsP_4_ metabolism by PPIP5K2 with an IC_50_ of 375 nM (Fig. 2A). We then obtained an X-ray structure of 5-PCF_2_Am-InsP_5_ (**1**) in complex with PPIP5K2 kinase domain and the stable ATP analogue AMPPNP. The structure shows that 5-PCF_2_Am-InsP_5_ binds to the catalytic site of PPIP5K2 in a similar orientation to 5-PP-InsP_5_ (Fig. 2B–D). While crystallography previously showed that 5-PA-InsP_5_ binds to two sites in PPIP5K2 (the catalytic site and a surface-mounted ligand capture site),^4^,^10^ electron density for **1** was seen only in the catalytic site (Fig. 2B). This is similar to the complex structures of PPIP5K2 with 5-PCP-InsP_5_ (ref. 7) or with 5-PP-InsP_5_ itself.^25^

The electron density for **1** (Fig. 2C) clearly shows that the planar amide unit adopts the *Z* conformation, and with the amide NH antiperiplanar to H-5 of *myo*-inositol. The carbonyl oxygen of the amide is orientated similarly to one non-bridging oxygen atom of the α-phosphate of bound 5-PP-InsP_5_ (Fig. 2E and F). Additionally, one of the fluorine atoms in the CF_2_ group stretches its position to that of the other non-bridging O-atom of an α-phosphate, which is expected to bring the negative charge distribution in this region closer to that of 5-PP-InsP_5_ than occurs with the PA analogue. The terminal phosphonate group in bound **1** is held in an extended conformation (Fig. 2E) although its orientation is different to that of the corresponding β-phosphate group in 5-PP-InsP_5_ bound to PPIP5K2 (Fig. 2F).

## Conclusions

We have described the design and synthesis of a novel type of stable PP-InsP analogue containing a phosphono-difluoroacetamide (PCF_2_Am) structure, intended to mimic the 5-diphosphate (PP) group of 5-PP-InsP_5_. As proof of principle, we examined the interaction of the new analogue, 5-PCF_2_Am-InsP_5_ (**1**), with the catalytic domain of the diphosphoinositol pentakisphosphate kinase PPIP5K2. A crystallographic study showed that **1** binds to the PP-InsP-binding site of the PPIP5K2 kinase domain in a similar orientation to that of the natural substrate 5-PP-InsP_5_ and that the PCF_2_Am structure can mimic several functionally important aspects of the diphosphate group (PP) in 5-PP-InsP_5_.

Recent years have seen a rapid increase in reported functions of PP-InsPs, the identification of their target proteins, and the characterisation of metabolising enzymes. Stabilised analogues of PP-InsPs have often played valuable roles in this work. No single analogue of a PP-InsP can perfectly mimic its steric and electronic features in all environments, and a range of compounds with complementary properties therefore offers a more versatile analytical approach. It is in this context that we propose that 5-PCF_2_Am-InsP_5_ (**1**) brings new opportunities. For example, the presence of the CF_2_ group in **1** means that its terminal phosphonate group should be more acidic than the equivalent group in 5-PA-InsP_5_ or 5-PCP-InsP_5_, making it more similar in this respect to the β-phosphate of 5-PP-InsP_5_ and potentially allowing stronger interactions with protein binding sites. In addition, the rigidity of the amide unit in **1** means that the PCF_2_Am unit contains fewer rotatable bonds than the equivalent PP, PA or PCP structures. A more rigid ligand is less likely to lose entropy on binding than a more flexible one, and thus may bind more tightly to some sites. Note also that the 5-diphosphate in 5-PP-InsP_5_ participates in complexing a hydrated Mg^2+^ ion, a property that is mimicked in the complex of 5-PCP-InsP_5_ with PPIP5K2.^7^ This Mg^2+^ ion is not present in the complex with **1** (Fig. 2E). Thus, 5-PCF_2_Am-InsP_5_ may be particularly useful for studying ligand/protein interactions that do not involve Mg^2+^.^9^ To the best of our knowledge, the PCF_2_Am group has not been previously employed as a diphosphate mimic; given the large number of biomolecules that contain diphosphate or polyphosphate motifs, it may find wider applications than those outlined here.

## Experimental

### General chemistry methods

All reagents and solvents were of commercial quality and were used without further purification. Petroleum ether used for chromatography and crystallisations was of fractions 40–60 °C. Alcohol **3** was synthesised as previously reported.^22^ Diethyl phosphonodifluoroacetic acid was synthesised according to a literature procedure.^24^ Thin-layer chromatography (TLC) was performed on pre-coated aluminium plates (Merck, silica gel 60, F254). Chromatograms were visualised under UV light and by dipping plates into either phosphomolybdic acid in EtOH, vanillin in acidic EtOH, or alkaline aqueous KMnO_4_, followed by heating. Flash column chromatography was performed on an ISCO CombiFlash Rf automated flash chromatography system using RediSep Rf disposable flash columns. NMR spectra were recorded on Bruker Avance III 400 and 500 MHz NMR spectrometers. Proton chemical shifts are reported in ppm (*δ*) relative to internal tetramethylsilane (TMS, 0.0 ppm) or with the solvent resonance relative to TMS employed as the internal standard (D_2_O, 4.79 ppm). The following abbreviations are used to describe resonances: br, broad; s, singlet; d, doublet; dd, double doublet; q, quartet; m, multiplet; t, triplet. ^13^C chemical shifts are reported relative to internal TMS (TMS, 0.0 ppm) or with the solvent resonance relative to TMS employed as the internal standard. The assignments of the proton and carbon atoms are based on 2D-NMR experiments (^1^H–^1^H-COSY, HSQC). ^31^P chemical shifts are reported in ppm (*δ*) relative to an 85% H_3_PO_4_ external standard (H_3_PO_4_, 0.0 ppm). ^19^F chemical shifts are reported in ppm (*δ*) relative to a CFCl_3_ external standard (CFCl_3_, 0.0 ppm). Melting points were determined using a Stanford Research Systems Optimelt MPA100 automated melting point system and are uncorrected. High resolution time-of-flight mass spectra were obtained on a Bruker Daltonics micrOTOF mass spectrometer using electrospray ionisation (ESI†).

### 2-*O*-Acetyl-5-deoxy-5-azido-1,6:3,4-bis-[*O*-(2,3-dimethoxybutane-2,3-diyl)]-*myo*-inositol (**4**)

To a stirred solution of alcohol **3** (1.15 g, 2.55 mmole) in dry dichloromethane (10 mL) and dry pyridine (1 mL) under N_2_ at –78 °C was added trifluoromethanesulfonic anhydride (4.0 mL of a 1.0 mol dm^–3^ solution in dichloromethane, 4.0 mmol) dropwise over 15 min. The cooling bath was removed and the solution was allowed to warm to room temperature. Stirring was continued overnight (16 h) after which time TLC (ethyl acetate : petroleum ether 2 : 1) showed complete conversion of alcohol (*R*_f_ 0.32) into a less polar product (*R*_f_ 0.70). Excess triflic anhydride was destroyed by careful addition of deionised water, and the mixture was diluted with dichloromethane (30 mL). The organic layer was separated and washed with 1.0 mol dm^–3^ HCl, saturated NaHCO_3_ and brine (40 mL of each), then dried over MgSO_4_ and concentrated under reduced pressure (no heat) to give crude triflate (1.45 g 2.49 mmole, 98%) as a reddish solid. To a solution of crude triflate (1.24 g, 2.13 mmol) in dry DMF (10 mL) at room temperature was added sodium azide (415 mg, 6.38 mmol). The mixture was stirred under N_2_ for 16 h, after which time TLC (ethyl acetate : petroleum ether 1 : 3) showed complete conversion of triflate (*R*_f_ 0.24, stains purple with vanillin) into a slightly less polar product (*R*_f_ 0.30, stains orange with vanillin). The solution was concentrated by evaporation under reduced pressure (care: explosion risk with azides!). The residue was taken up in ethyl acetate (50 mL) and the solution was washed with deionised water and brine (50 mL each), then concentrated. The residue was purified by flash chromatography on silica, eluting with ethyl acetate to give **4** as a white solid (892 mg, 1.88 mmol, 86% from **3**); crystals from boiling petroleum ether, m.p. 198–199 °C; ^1^H NMR (400 MHz, CDCl_3_) *δ* 1.23 (6H, s, 2 × CH_3_), 1.30 (6H, s, 2 × CH_3_), 2.12 (3H, s, CH_3_CO), 3.24 (6H, s, OCH_3_), 3.30 (6H, s, OCH_3_), 3.52 (1H, t, *J* 10.0 Hz, H-5), 3.66 (2H, dd, *J* 10.0, 2.6 Hz, H-1 and H-3), 3.85 (2H, t, *J* 10.0 Hz, H-4 and H-6), 5.41 (1H, t, *J* 2.7 Hz, H-2); ^13^C NMR (100 MHz, CDCl_3_) *δ* 17.41 (CH_3_), 17.62 (CH_3_), 21.10 (*C*H_3_CO), 48.00 (OCH_3_), 48.16 (OCH_3_), 61.04 (C-5), 67.53 (C-1 and C-3), 68.51 (C-4 and C-6), 68.94 (C-2), 99.72 (BDA quaternary C), 99.94 (BDA quaternary C), 170.23 (C=O); HRMS (*m*/*z*) [M + Na]^+^ calcd. for C_20_H_33_N_3_O_10_, 498.2058; found 498.2035.

### 2-*O*-Benzyl-5-deoxy-5-azido-1,6:3,4-bis-[*O*-(2,3-dimethoxybutane-2,3-diyl)]-*myo*-inositol (**5**)

To a solution of **4** (530 mg, 1.11 mmol) in THF (12 mL) was added a solution of LiOH·H_2_O (420 mg, 10 mmol) in deionised water (3 mL) and methanol (12 mL). The mixture was stirred vigorously at room temperature for 2 h, after which time TLC (ethyl acetate : petroleum ether 1 : 1) showed complete conversion of **4** (*R*_f_ 0.54) into a more polar product (*R*_f_ 0.28). The solution was concentrated and the residue was taken up in ethyl acetate (50 mL). The solution was washed with water (2 × 50 mL), dried (MgSO_4_) and concentrated to give the alcohol as a white solid (484 mg). This crude alcohol was taken up in DMF (10 mL). The solution was stirred at 0 °C and sodium hydride (60% suspension in mineral oil, 67 mg, 1.67 mmole) was added. After 30 min, benzyl bromide (0.16 mL, 1.3 mmol) was added and the mixture was stirred overnight (16 h) at room temperature. Excess NaH was destroyed by careful addition of water and the solution was concentrated. The residue was taken up in ethyl acetate (50 mL) and washed with water and brine (50 ml each), dried (MgSO_4_) and concentrated to give a solid residue. Purification by flash chromatography on silica (ethyl acetate in petroleum ether, 0 to 50%) gave **5** as a white solid (541 mg, 1.03 mmol, 93% over two steps); *R*_f_ 0.34 (ethyl acetate : petroleum ether 1 : 3); crystals from petroleum ether, m.p. 175–176.5 °C; ^1^H NMR (400 MHz, CDCl_3_) *δ* 1.31 (6H, s, 2 × CH_3_), 1.32 (6H, s, 2 × CH_3_), 3.23 (6H, s, OCH_3_), 3.29 (6H, s, OCH_3_), 3.49 (1H, t, *J* 10.0 Hz, H-5), 3.57 (2H, dd, *J* 10.0, 2.5 Hz, H-1 and H-3), 3.80 (1H, t, *J* 2.7 Hz, H-2), 4.03 (2H, t, *J* 10.1 Hz, H-4 and H-6), 4.84 (2H, s, OC*H*_2_Ph), 7.22–7.27 (1H, m, *para*-H of Ph), 7.29–7.34 (2H, m, *meta*-H of Ph), 7.48–7.51 (2H, m, *ortho*-H of Ph); ^13^C NMR (100 MHz, CDCl_3_) *δ* 17.61 (CH_3_), 17.70 (CH_3_), 47.92 (OCH_3_), 48.00 (OCH_3_), 61.52 (C-5), 68.46 (C-4 and C-6), 69.70 (C-1 and C-3), 73.94 (OCH_2_Ph), 76.02 (C-2), 99.51 (BDA quaternary C), 99.71 (BDA quaternary C), 127.02 (*para*-C of Ph), 127.62 (CH of Ph), 127.90 (CH of Ph), 139.49 (*ipso*-C of Ph); HRMS (*m*/*z*) [M + Na]^+^ calcd. for C_25_H_37_N_3_O_9_, 546.2422; found 546.2406.

### 1,2,3,4,6-Penta-*O*-benzyl-5-deoxy-5-azido-*myo*-inositol (**6**)

To a stirred solution of **5** (507 mg, 0.968 mmol) in dichloromethane (5 mL) was added 95% aqueous TFA (5 mL). A yellow colour (butanedione) appeared within 30 s. After 30 min, the solution was concentrated to leave the crude tetraol as a white solid (325 mg). This solid was taken up in dry DMF (10 mL) and the solution was cooled to 0 °C, before sodium hydride (60% suspension in mineral oil, 240 mg, 6.0 mmole) was added. The stirred suspension was allowed to warm to room temperature and then cooled again to 0 °C before benzyl bromide (0.60 mL, 5.1 mmol) was added, dropwise. The cooling bath was removed and the mixture was stirred overnight (16 h) at room temperature. Excess NaH was destroyed by careful addition of water and the solution was concentrated. The residue was taken up in dichloromethane (50 mL) and washed with water and brine (50 ml each), dried (MgSO_4_) and concentrated to give an oily residue. Purification by flash chromatography on silica (ethyl acetate in petroleum ether, 0 to 50%) gave **6** as a colourless oil, which slowly crystallised (576 mg, 0.878 mmol, 91% over two steps); *R*_f_ 0.40 (ethyl acetate : petroleum ether 1 : 4); crystals from boiling ethanol, m.p. 89.0–90.5 °C; ^1^H NMR (400 MHz, CDCl_3_) *δ* 3.33 (2H, dd, *J* 9.7, 2.2 Hz, H-1 and H-3), 3.35 (1H, t, *J* 9.9 Hz, H-5), 3.85 (2H, t, *J* 9.8 Hz, H-4 and H-6), 4.00 (1H, t, *J* 2.4 Hz, H-2), 4.57, 4.62 (4H, AB quartet, *J*_AB_ 11.7 Hz, 2 × OC*H*_2_Ph), 4.81, 4.87 (4H, AB quartet, *J*_AB_ 10.5 Hz, 2 × OC*H*_2_Ph), 4.85 (2H, s, OC*H*_2_Ph), 7.25–7.41 (25H, m, Ph); ^13^C NMR (100 MHz, CDCl_3_) *δ* 67.68 (C-5), 72.77 (2 × OCH_2_Ph), 74.25 (OCH_2_Ph), 74.31 (C-2), 75.73 (2 × OCH_2_Ph), 79.77 (C-4 and C-6), 81.20 (C-1 and C-3), 127.46, 127.57, 127.70, 127.72, 127.78, 128.20, 128.30, 128.34 and 128.41 (CH of Ph), 138.12 (2 × *ipso*-C of Ph), 138.24 (2 × *ipso*-C of Ph), 138.79 (*ipso*-C of Ph); HRMS (*m*/*z*) [M + Na]^+^ calcd. for C_41_H_41_N_3_O_5_, 678.2938; found 678.2966.

### 1,2,3,4,6-Penta-*O*-benzyl-5-deoxy-5-amino-*myo*-inositol (**7**)

To dry THF (3 mL) under argon was added LiAlH_4_ (1.0 mL of a 1.0 mol dm^–3^ solution in THF, 1.0 mmol). The solution was stirred at 0 °C under argon and a solution of the azide **6** (420 mg, 0.640 mmol) in dry THF (4 mL) was injected over 10 min. After 30 min, the cooling bath was removed and the solution was stirred at room temperature for a further 1 h. The solution was then cooled to 0 °C again and excess LiAlH_4_ was destroyed by addition of ethyl acetate (2 mL). The solution was allowed to warm to room temperature and then diluted with water (20 mL). The suspension was extracted with diethyl ether (3 × 20 mL). The aqueous layer was re-extracted with dichloromethane (20 mL) and the combined organic extracts were then washed with brine (50 mL), dried (MgSO_4_) and concentrated to give amine **7** as a white solid (402 mg, 0.638 mmol, 100%); ^1^H NMR (400 MHz, CDCl_3_) *δ* 1.73 (2H, broad s, NH_2_), 2.81 (1H, t, *J* 9.6 Hz, H-5), 3.40 (2H, dd, *J* 9.6, 2.2 Hz, H-1 and H-3), 3.85 (2H, t, *J* 9.6 Hz, H-4 and H-6), 4.08 (1H, t, *J* 2.4 Hz, H-2), 4.61 (4H, s, OC*H*_2_Ph), 4.69, 5.01 (4H, AB quartet, *J*_AB_ 11.0 Hz, 2 × OC*H*_2_Ph), 4.88 (2H, s, OC*H*_2_Ph), 7.25–7.43 (25H, m, Ph); ^13^C NMR (100 MHz, CDCl_3_) *δ* 56.17 (C-5), 72.41 (2 × OCH_2_Ph), 74.16 (OCH_2_Ph), 74.26 (C-2), 75.61 (2 × OCH_2_Ph), 81.66 (C-4 and C-6), 82.23 (C-1 and C-3), 127.27, 127.57, 127.63, 127.65, 127.99, 128.08, 128.12, 128.40 and 128.41 (CH of Ph), 138.33 (2 × *ipso*-C of Ph), 138.85 (2 × *ipso*-C of Ph), 139.09 (*ipso*-C of Ph). HRMS (*m*/*z*) [M + H]^+^ calcd. for C_41_H_43_NO_5_, 630.3214; found 630.3210.

### 1,2,3,4,6-Penta-*O*-benzyl-5-deoxy-5-(diethylphosphono-difluoroacetamido)-*myo*-inositol (**8**)

To a solution of amine **7** (141 mg, 0.224 mmol) and EDAC (86 mg, 0.448 mmol) in dry dichloromethane (3 mL) under N_2_ was added a solution of diethyl phosphonodifluoroacetic acid^24^ (104 mg, 0.448 mmol) in dry dichloromethane (2 mL). The solution was stirred at room temperature for 1 h, after which time TLC (ethyl acetate : petroleum ether 1 : 1) showed total conversion of amine (streak, ∼*R*_f_ 0.20) into a less polar product (spot, *R*_f_ 0.46). Ethyl acetate (30 mL) was added and the solution was washed with saturated NaHCO_3_, 1.0 mol dm^–3^ HCl and brine (20 mL each), then dried (MgSO_4_) and concentrated. The residue was purified by flash chromatography on silica (ethyl acetate in petroleum ether, 0 to 100%) to give **8** as a colourless oil, which slowly crystallised (164 mg, 0.194 mmol, 87%); *R*_f_ 0.40 (ethyl acetate : petroleum ether 1 : 4); crystals from boiling diisopropyl ether, m.p. 132.0–133.5 °C; ^1^H NMR (400 MHz, CDCl_3_) *δ* 1.24 (6H, td, *J* 7.1, 0.6 Hz, POCH_2_C*H*_3_), 3.46 (2H, dd, *J* 9.0, 2.2 Hz, H-1 and H-3), 3.98 (2H, t, *J* 9.1 Hz, H-4 and H-6), 4.03 (1H, t, *J* 2.2 Hz, H-2), 4.10 (1H, q, *J* 9.2 Hz, H-5), 4.13–4.21 (4H, m, POC*H*_2_CH_3_), 4.56, 4.60 (4H, AB quartet, *J*_AB_ 11.8 Hz, 2 × OC*H*_2_Ph), 4.73, 4.80 (4H, AB quartet, *J*_AB_ 11.8 Hz, 2 × OC*H*_2_Ph), 4.84 (2H, s, OC*H*_2_Ph), 6.66 (1H, d, *J* 9.2 Hz, NH), 7.20–7.33 (23H, m, Ph), 7.39–7.42 (2H, m, Ph); ^13^C NMR (100 MHz, CDCl_3_) *δ* 16.22 (^3^*J*_CP_ 5.6 Hz, POCH_2_*C*H_3_), 54.68 (C-5), 65.65 (^2^*J*_CP_ 6.6 Hz, PO*C*H_2_CH_3_), 72.77 (2 × OCH_2_Ph), 74.12 (OCH_2_Ph), 74.55 (C-2), 74.76 (2 × OCH_2_Ph), 78.31 (C-4 and C-6), 81.34 (C-1 and C-3), 111.92 (td, ^1^*J*_CF_ 271.6 Hz, ^1^*J*_CP_ 197.9 Hz, CF_2_), 127.40, 127.49, 127.66, 127.69, 127.83, 127.92, 128.19, 128.23 and 128.36 (CH of Ph), 138.11 (2 × *ipso*-C of Ph), 138.35 (2 × *ipso*-C of Ph), 138.77 (*ipso*-C of Ph), 161.40 (td, ^2^*J*_CF_ 24.6 Hz, ^2^*J*_CP_ 17.3 Hz, C=O), ^31^P NMR (CDCl_3_, 162 MHz, ^1^H-decoupled) *δ* 3.47 (1 P, t, ^2^*J*_PF_ 96.1 Hz); ^19^F NMR (CDCl_3_, 471 MHz) *δ* –116.11 (2 F, d, ^2^*J*_FP_ 96.1 Hz); HRMS (*m*/*z*) [M]^–^ calcd. for C_47_H_52_F_2_NO_9_P, 842.3275; found 842.3264.

### 5-Deoxy-5-(diethylphosphonodifluoroacetamido)-*myo*-inositol (**9**)

To a solution of **8** (130 mg, 0.154 mmol) in methanol (8 mL), THF (2 mL), deionised water (2 mL) and acetic acid (1 mL) was added palladium hydroxide on activated charcoal (20%, 50% water, 50 mg). The suspension was shaken in a Parr hydrogenator under H_2_ (50 p.s.i.) for 24 h. The catalyst was removed by filtration through a PTFE syringe filter and the resulting colourless solution was concentrated, then dried under vacuum to give pentaol **9** as a white solid (61 mg, 0.154 mmol, 100%); TLC (dichloromethane : methanol 2 : 1): *R*_f_ 0.40; ^1^H NMR (400 MHz, D_2_O) *δ* 1.38 (6H, broad t, *J* ∼7 Hz, POCH_2_C*H*_3_), 3.61 (2H, broad d, *J* ∼9 Hz, H-1 and H-3), 3.71–3.81 (3H, m, H-4, H-5 and H-6), 4.10 (1H, broad s, H-2), 4.35–4.42 (4H, m, POC*H*_2_CH_3_); ^13^C NMR (100 MHz, D_2_O) *δ* 15.64 (^3^*J*_CP_ 5.2 Hz, POCH_2_*C*H_3_), 56.28 (C-5), 67.37 (^2^*J*_CP_ 7.1 Hz, PO*C*H_2_CH_3_), 70.05 (C-4 and C-6), 71.91 (C-2), 72.04 (C-1 and C-3), 111.71 (td, ^1^*J*_CF_ 271.8 Hz, ^1^*J*_CP_ 208.7 Hz, CF_2_), 163.33 (td, ^2^*J*_CF_ 24.5 Hz, ^2^*J*_CP_ 16.2 Hz, C=O); ^31^P NMR (162 MHz, D_2_O, ^1^H-decoupled) *δ* 4.39 (t, ^2^*J*_PF_ 101.5 Hz); ^19^F NMR (471 MHz, D_2_O) *δ* –117.62 (d, ^2^*J*_FP_ 101.4 Hz); HRMS (*m*/*z*) [M + Na]^+^ calcd. for C_12_H_22_F_2_NO_9_P, 416.0892; found 416.0876.

### 5-Deoxy-5-(diethylphosphonodifluoroacetamido)-*myo*-inositol 1,2,3,4,6-*O*-pentakis(dibenzylphosphate) (**10**)

To a stirred suspension of pentaol **9** (30 mg, 0.076 mmol) and 5-phenyl-1*H*-tetrazole (83 mg 0.57 mmol) in dry dichloromethane (3 mL) under N_2_ at room temperature was added bis(benzyloxy)diisopropylaminophosphine (0.20 mL, 0.60 mmol). The mixture was stirred under N_2_ at room temperature for 3 h and then cooled to –78 °C, before *m*CPBA (70%, 187 mg, 0.760 mmol) was added. The mixture was allowed to warm to room temperature and then diluted with EtOAc (30 mL). The clear, colourless solution was washed with 10% aq. Na_2_SO_3_ solution (3 × 25 mL), dried over MgSO_4_ and concentrated. The residue was purified by flash chromatography (EtOAc in petroleum ether, 0 to 100%) to give **10** as a colourless oil (85 mg, 0.050 mmole, 66%); TLC (EtOAc : petroleum ether, 4 : 1): *R*_f_ 0.56; ^1^H NMR (CDCl_3_, 400 MHz) *δ* 1.27 (6H, td, *J* 7.1, 0.5 Hz, POCH_2_C*H*_3_), 4.20–4.33 (5H, m, 2 × POC*H*_2_CH_3_ and H-5), 4.39 (2H, broad t, *J* ∼9.5 Hz, H-1 and H-3), 4.82 (2H, q, *J* ∼10 Hz, H-4 and H-6), 4.89–5.11 (18H, POC*H*_2_Ph), 5.15–5.19 (2H, POC*H*_2_Ph), 5.63 (1H, broad d, *J* ∼9 Hz, H-2), 7.11–7.28 (50H, m, Ph), 7.52 (1H, broad d, *J* ∼9 Hz, NH); ^13^C NMR (101 MHz, CDCl_3_) *δ* 16.31 (d, ^3^*J*_CP_ 5.6 Hz, POCH_2_*C*H_3_), 52.70 (broad, C-5), 65.35 (d, ^2^*J*_CP_ 6.4 Hz, PO*C*H_2_CH_3_), 69.75–70.06 (overlapping signals with *J*_CP_ couplings, PO*C*H_2_Ph), 73.72 (with *J*_CP_ couplings, C-4 and C-6), 74.28 (with *J*_CP_ couplings, C-1 and C-3), 75.93 (with *J*_CP_ couplings, C-2), 112.1 (^1^*J*_CP_ 205 Hz, ^1^*J*_CF_ unreadable through noise, CF_2_), 127.83, 127.95, 128.12, 128.16, 128.21, 128.26, 128.32, 128.36, 128.39, 128.45, 128.49 (*C*H of Ph), 135.54–135.74 (overlapping signals with *J*_CP_ couplings, *ipso*-C of Ph), 162.85 (^2^*J*_CP_ ∼18 Hz, ^2^*J*_CF_ unreadable through noise, C=O); ^31^P NMR (162 MHz, CDCl_3_, ^1^H-decoupled) *δ* –2.30 (1 P, P-2), –1.64 (2 P), –0.11 (2 P), 3.20 (1 P, t, ^2^*J*_PF_ 93.6 Hz); ^19^F NMR (471 MHz, CDCl_3_) *δ* –113.48 (d, ^2^*J*_FP_ 93.4 Hz); HRMS (*m*/*z*) [M + Na]^+^ calcd. for C_82_H_87_F_2_NO_24_P_6_, 1716.3904; found 1716.3965; [M + H]^+^ calcd. for C_82_H_87_F_2_NO_24_P_6_, 1694.4085; found 1694.4130.

### 5-Deoxy-5-(phosphonodifluoroacetamido)-*myo*-inositol 1,2,3,4,6-pentakisphosphate (**1**)

A stirred solution of **10** (68 mg, 40 μmole) in dry dichloromethane (2 mL) was cooled to 0 °C under N_2_ and trimethylsilyl bromide (1 mL) was added dropwise over 5 min. The solution was allowed to warm gradually to room temperature, and stirring was continued for 48 h. The solution was concentrated and methanol (5 mL) was added to the residue. The resulting colourless solution was stirred at room temperature for a further 1 h, and then concentrated to give a white gum. The gum was washed with diethyl ether (3 × 2 mL), then taken up in aqueous triethylammonium bicarbonate (1.0 mol dm^–3^, pH 7.6, 5 mL). This solution was then washed with diethyl ether (3 × 5 mL) and concentrated. The residue was re-dissolved in MilliQ water and lyophilised to give the triethylammonium salt of the title compound **1** as a colourless solid (47 mg, 34 μmole, 85%); ^1^H NMR (500 MHz, D_2_O) *δ* 1.28 (approx. 57 H, t, *J* 7.3 Hz, CH_3_ of TEA^+^), 3.20 (approx. 38 H, q, *J* 7.3 Hz, CH_2_ of TEA^+^), 4.16 (1H, broad t, *J* ∼10 Hz, H-5), 4.30 (2H, tt, *J* 9.6, 2.0 Hz, H-1 and H-3), 4.55 (2H, q, *J* 9.7 Hz, H-4 and H-6), 4.87 (1H, dt, *J* 9.8, 2.4 Hz, H-2); ^13^C NMR (126 MHz, D_2_O) *δ* 8.19 (CH_3_ of TEA^+^), 46.54 (CH_2_ of TEA^+^), 53.91 (C-5), 74.44 (C-1, C-3, C-4 and C-6), 75.86 (C-2), 114.65 (dt, ^1^*J*_CP_ 176.6 Hz, ^1^*J*_CF_ 268.7 Hz, CF_2_), 166.30 (td, ^2^*J*_CF_ 25.3 Hz, ^2^*J*_CP_ 15.3 Hz, C=O); ^31^P NMR (162 MHz, D_2_O, ^1^H-decoupled) *δ* –0.75 (1 P, P-2), –0.32 (2 P), 0.15 (2 P), 0.64 (1 P, t, ^2^*J*_PF_ 87.9 Hz, P-5); ^19^F NMR (471 MHz, D_2_O) *δ* –118.86 (d, ^2^*J*_FP_ 85.5 Hz, CF_2_); HRMS (*m*/*z*) [M – H]^–^ calcd. for C_8_H_19_F_2_NO_24_P_6_, 735.8618; found 735.8654.

### Protein expression, purification, crystallisation and structure determination

The kinase domain of human PPIP5K2 (residues 41–366) was sub-cloned, expressed and purified as before.^25^ PPIP5K2 kinase domain was crystallised by hanging drop vapour diffusion against a well buffer of 12% (w/v) PEG 3350, 20 mM MgCl_2_, 0.1 M HEPES, pH 7.0, 2 mM CdCl_2_, 1 mM AMPPNP at 4 °C. The crystals were transferred to a stabilising buffer containing 22% (w/v) PEG 3350, 10 mM MgCl_2_, 0.1 M sodium acetate, pH 5.2 at 4 °C and the crystals were then soaked under the above stabilising buffer for three days with 2 mM compound **1**. Cryosolvent was prepared by adding 33% ethylene glycol into the soaking buffer. Diffraction data were collected using APS beamlines 22-ID. All data were processed with the program HKL2000. The structure was determined using rigid body and direct Fourier synthesis, and refined with the equivalent and expanded test sets. The structure was further manually rebuilt with COOT and refined with REFMAC from the CCP4 package. Ligand topology and parameter files were prepared using the PRODRG server. The molecular graphics representations were prepared with the program PyMol (Schrödinger, LLC). Atomic coordinates and structure factors have been deposited with the Protein Data Bank with accession code ; 6N5C.

### PPIP5K2 kinase assay

Human PPIP5K2 kinase domain (residues 1–366, 2.5 μg mL^–1^) was incubated at 24 °C for 30 min with buffer containing 20 mM Tris–HCl, pH 7.5, 10 mM MgCl_2_, 0.1 mM ADP, 100 nM 1,5-[PP]_2_-InsP_4_ and various concentration of compound **1** in a 20 μL assay. The generated ATP was measured using a Molecular Probes ATP Determination kit (Thermo Fisher Scientific catalog number A22066). The IC_50_ value for **1** was calculated using GraphPad Prism.

## Conflicts of interest

There are no conflicts to declare.

## Supplementary Material

Supplementary information

## References

[cit1] Shears S. B. (2018). J. Cell. Physiol..

[cit2] Thomas M. P., Potter B. V. L. (2014). FEBS J..

[cit3] Riley A. M., Wang H., Weaver J. D., Shears S. B., Potter B. V. L. (2012). Chem. Commun..

[cit4] Riley A. M., Wang H., Shears S. B., Potter B. V. L. (2015). Chem. Commun..

[cit5] Wu M., Dul B. E., Trevisan A. J., Fiedler D. (2013). Chem. Sci..

[cit6] Wu M., Chong L. S., Capolicchio S., Jessen H. J., Resnick A. C., Fiedler D. (2014). Angew. Chem., Int. Ed..

[cit7] Hager A., Wu M., Wang H., Brown N. W., Shears S. B., Veiga N., Fiedler D. (2016). Chem. – Eur. J..

[cit8] Brown N. W., Marmelstein A. M., Fiedler D. (2016). Chem. Soc. Rev..

[cit9] Wu M., Chong L. S., Perlman D. H., Resnick A. C., Fiedler D. (2016). Proc. Natl. Acad. Sci. U. S. A..

[cit10] Wang H., Godage H. Y., Riley A. M., Weaver J. D., Shears S. B., Potter B. V. L. (2014). Chem. Biol..

[cit11] Vertuani S., Baldisserotto A., Varani K., Borea P. A., De Marcos Maria Cruz B., Ferraro L., Manfredini S., Dalpiaz A. (2012). Eur. J. Med. Chem..

[cit12] Elliott T. S., Slowey A., Ye Y., Conway S. J. (2012). Med. Chem. Commun..

[cit13] Blackburn G. M., England D. A., Kolkmann F. (1981). J. Chem. Soc., Chem. Commun..

[cit14] O'Hagan D., Rzepa H. S., Romanenko V. D., Kukhar V. P., Panigrahi K., Nelson D. L., Berkowitz D. B., Ivanova M. V., Bayle A., Besset T., Pannecoucke X., Poisson T. (1997). Chem. Commun..

[cit15] Combs A. P., Bahta M., Lountos G. T., Dyas B., Kim S. E., Ulrich R. G., Waugh D. S., Burke T. R. (2010). J. Med. Chem..

[cit16] Kobzar O. L., Shevchuk M. V., Lyashenko A. N., Tanchuk V. Y., Romanenko V. D., Kobelev S. M., Averin A. D., Beletskaya I. P., Vovk A. I., Kukhar V. P. (2015). Org. Biomol. Chem..

[cit17] Blackburn G. M., Kent D. E., Kolkmann F., Baszczyňski O., Janeba Z., Romanenko V. D., Kukhar V. P. (1984). J. Chem. Soc., Perkin Trans. 1.

[cit18] Shears S. B. (2016). Biochem. Soc. Trans..

[cit19] Pajkert R., Milewska M., Röschenthaler G. V., Koroniak H. (2009). J. Fluorine Chem..

[cit20] Blackburn G. M., Jakeman D. L., Ivory A. J., Williamson M. P., Jakeman D. L., Ivory A. J., Williamson M. P., Blackburn G. M., Kotsikorou E., Sahota G., Oldfield E. (1994). Bioorg. Med. Chem. Lett..

[cit21] Grison C., Coutrot P., Comoy C., Balas L., Joliez S., Lavecchia G., Oliger P., Penverne B., Serre V., Hervé G. (2004). Eur. J. Med. Chem..

[cit22] Riley A. M., Jenkins D. J., Potter B. V. L. (1998). Carbohydr. Res..

[cit23] Montchamp J. L., Tian F., Hart M. E., Frost J. W., Baeschlin D. K., Chaperon A. R., Green L. G., Hahn M. G., Ince S. J., Ley S. V. (1996). J. Org. Chem..

[cit24] Hamilton C. J., Roberts S. M. (1999). J. Chem. Soc., Perkin Trans. 1.

[cit25] Wang H., Falck J. R., Hall T. M., Shears S. B. (2012). Nat. Chem. Biol..

[cit26] Ravi A., Hassan S. Z., Vanikrishna A. N., Sureshan K. M. (2017). Chem. Commun..

