# Supplementary material for: Synthesis of an α-phosphono-α,α-difluoroacetamide analogue of the diphosphoinositol pentakisphosphate 5-InsP7
Source: Medchemcomm. 2019 Jun 7;10(7):1165–72. doi: 10.1039/c9md00163h (PMC6657673; doi:10.1039/c9md00163h)

Electronic Supplementary Material (ESI) for MedChemComm.
This journal is © The Royal Society of Chemistry 2019

Supplementary Information

Synthesis of an α-phosphono-α,α-difluoroacetamide analogue of the diphosphoinositol pentakisphosphate 5-$InsP_7$

Andrew M. Riley,[a] Huanchen Wang,[b] Stephen B. Shears and Barry V. L. Potter[a]

[a]*Medicinal Chemistry & Drug Discovery, Department of Pharmacology, University of Oxford, Mansfield Road, Oxford OX1 3QT.*

[b]*Inositol Signaling Group, Laboratory of Signal Transduction, National Institute of Environmental Health Sciences, National Institutes of Health, Research Triangle Park, North Carolina, USA*

Table of Contents

Data collection and structure refinement statistics S2

Figure S1: Effect of CF_2 on ^{31}P NMR spectrum of **1** S3

Synthesis of 5-PCH_2Am-$InsP_5$ (**1a**) S4-S7

NMR Spectra for all compounds S8-S25

Data collection and structure refinement statistics

Compound ID	**1**
PDB Accession IDs	6N5C
**Data collection**	
Resolution (Å)*	50-1.95 (1.98)
Space group	$P\ 2_1\ 2_1\ 2_1$
Cell dimensions *a*, b, *c* (Å)	88.60 111.03 41.43
R_{meas} *	0.098(0.835)
CC1/2 in the highest shell	0.820
I/σI*	23.38(2.63)
Completeness (%)*	96.87 (95.45)
Redundancy *	6.1(6.1)
**Refinement**	
Resolution(Å)*	1.95(2.00)
R_{work} *	0.175 (0.220)
R_{free}*	0.214 (0.262)
No. atoms Protein	2539
No. atoms Ligands	108
No. atoms Solvent	296
B-factors ($Å^2$) Overall	34.15
B-factors (Å2) Protein	32.79
B-factors (Å2) Ligands	43.77
B-factors (Å2) Solvent	42.30
R.m.s.d. Bond length(Å)	0.008
R.m.s.d. Bond Angle (°)	1.563
Ramachandran favored (%)	96.83
Ramachandran allowed (%)	2.86
Ramachandran outliers (%)	0.32
Rotamer outliers (%)	1.42
Clash score	2.30

*The numbers in parentheses are for the highest resolution shell.

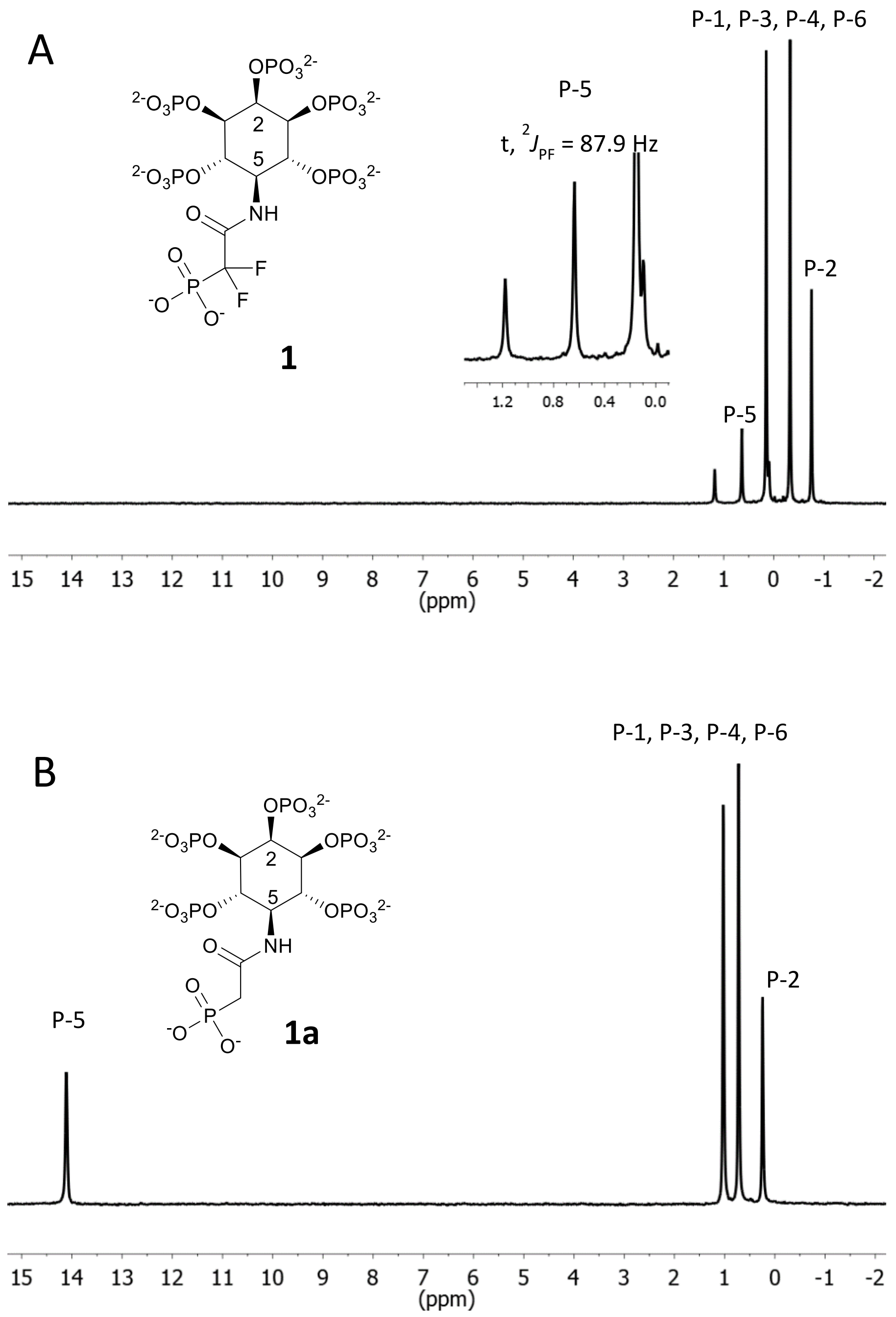

Figure S1. ^{31}P NMR spectra of 5-PCF$_2$Am-InsP$_5$ (**1**) and 5-PCH$_2$Am-InsP$_5$ (**1a**) (triethylammonium salts in D_2O, 162 MHz, ^1H-decoupled). The P-5 signal in **1** appears as a triplet, due to phosphorus-fluorine coupling. It has previously been reported[1] that the pKas for the second dissociation of phosphonic acids correlate well with the ^{31}P NMR shift of the phosphonate P atom. Thus, the upfield shift of P-5 in **1** is consistent with increased acidity of its phosphonate group compared to the phosphonate in **1a**. For the synthesis of **1a**, see below.

1. D. L. Jakeman, A. J. Ivory, M. P. Williamson and G. M. Blackburn, *J. Med. Chem.*, 1998, **41**, 4439-4452.

Synthesis of 5-deoxy-5-(phosphonoacetamido)-*myo*-inositol 1,2,3,4,6-pentakisphosphate (1a)

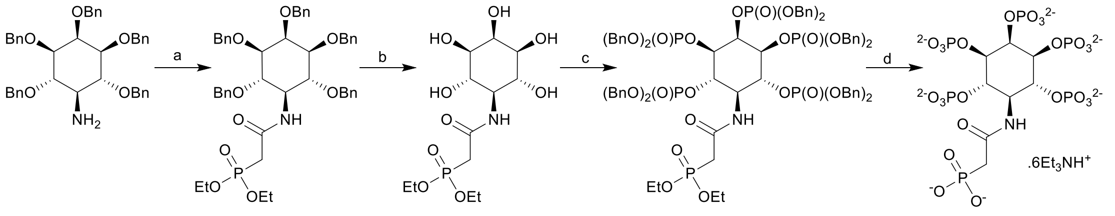

7 **8a** **9a** **10a** **1a**

Scheme. Synthesis of 5-PCH_2Am-InsP_5 (**1a**). Reagents and conditions: a. $(EtO)_2P(O)CH_2COOH$, EDAC, CH_2Cl_2, 79%; b. H_2 (50 p.s.i.) $Pd(OH)_2$/C, MeOH, THF, AcOH, 80%; c. i. $(BnO)_2PN^iPr_2$, 5-phenyl-1*H*-tetrazole, CH_2Cl_2; ii. *m*CPBA, 67%; d. i. TMSBr, CH_2Cl_2; ii. MeOH; iii. aqueous triethylammonium bicarbonate, 85%. Bn, benzyl.

Compound **1a**, the non-fluorinated equivalent of compound **1** was synthesised by reaction of amine **7** with commercially available diethyl phosphonoacetic acid in the presence of EDAC, giving phosphonoacetamide **8a**. The five benzyl protecting groups were then removed by hydrogenolysis over $Pd(OH)_2$ on carbon to give pentaol **9a**. When NMR data of **9a** were obtained in D_2O, the ^1H NMR spectrum (see data below) showed that the CH_2 protons of the phosphonoacetamide group had partially exchanged with deuterium from D_2O over the time course of the NMR experiments. When this sample was used in the next step (phosphorylation), the deuterium was retained in the product **10a**. After deprotection to give **1a**, the extent of deuterium incorporation present did not change, even though the NMR sample of **1a** in D_2O was kept for several days. This suggest that, even though deuterium exchange took place for the ethyl protected phosphonoacetamide in pentaol **9a**, and was retained in **10a**, no further exchange occurred in the deprotected product **1a**, in which the phosphonate group is ionised and presumably any required enolisation is disfavoured. Indeed, mass spectrometry (see data below) showed that samples of **1a** remained deuterated, even though **1a** had been left to stand in (undeuterated) milliQ water, lyophilised, dissolved in milliQ water once more, distributed into vials and concentrated in a vacuum centrifuge over several hours. Attempts to exchange the deuterium in samples of **1a** back to hydrogen by leaving samples in aqueous solution at high or low pH for extended periods have so far been unsuccessful. The incorporation of deuterium into the phosphonoacetamide unit of **1a** may suggest strategies for developing tritiated versions of **1a** and related analogues of PP-InsPs that may be useful in distinguishing potential receptor-mediated roles for such PP-InsPs in contrast to those involving phosphoryl transfer.

1,2,3,4,6-penta-*O*-benzyl-5-deoxy-5-(diethylphosphonoacetamido)-*myo*-inositol (8a). To a solution of amine **7** (178 mg, 0.283 mmol) and EDAC (82 mg, 0.428 mmol) in dry dichloromethane (3 mL) under N_2 was added a solution of diethyl phosphonoacetic acid (0.1 mL, 0.6 mmol) in dry dichloromethane (2 mL). The solution was stirred at room temperature for 3 h, after which time TLC (dichloromethane: methanol: triethylamine 1:1:1) showed total conversion of amine (R_f 0.23) into a slightly more polar product (R_f 0.13). Dichloromethane (15 mL) was added and the solution was washed with saturated $NaHCO_3$, 1.0 M, HCl and brine (15 mL each), then dried ($MgSO_4$) and concentrated. The residue was purified by flash chromatography on silica (ethyl acetate in petrol, 0 to 100%) to give **8a** as a white solid (181 mg, 0.224 mmol, 79%); R_f 0.30 (ethyl acetate:petrol 2:1); crystals from boiling diisopropyl ether, m.p. 134–135 °C; ^{1}H NMR (400 MHz, $CDCl_3$) δ 1.11 (6 H, t, J 7.1, $POCH_2CH_3$), 2.57 (2 H, d, $^2J_{HP}$ 20.4 Hz, PCH_2), 3.42 (2 H, dd, J 9.3, 2.3 Hz, H-1 and H-3), 3.83–3.98 (6 H, m, $POCH_2CH_3$, H-4 and H-6), 4.02 (1 H, t, J 2.3 Hz, H-2), 4.10 (1 H, q, J 9.8 Hz, H-5), 4.54, 4.58 (4 H, AB quartet, J_{AB} 11.7 Hz, 2 × OCH_2Ph), 4.71, 4.85 (4 H, AB quartet, J_{AB} 11.5 Hz, 2 × OCH_2Ph), 4.86 (2 H, s, OCH_2Ph), 6.64 (1 H, d, J 9.3 Hz, NH), 7.20–7.33 (23 H, m, Ph), 7.40–7.42 (2 H, m, Ph); ^{13}C NMR (101 MHz, $CDCl_3$) δ 16.11 ($^3J_{CP}$ 6.0 Hz, $POCH_2CH_3$), 34.89 (d, $^1J_{CP}$ 129.6 Hz, CH_2P), 54.28 (C-5), 62.55 ($^2J_{CP}$ 6.4 Hz, $POCH_2CH_3$), 72.72 (2 × OCH_2Ph), 74.11 (OCH_2Ph), 74.54 (C-2), 74.66 (2 × OCH_2Ph), 79.33 (C-4 and C-6), 81.46 (C-1 and C-3), 127.22, 127.41, 127.60, 127.63, 127.67, 127.87, 128.20, and 128.33 (*C*H of Ph), 138.26 (2 × *ipso-C* of Ph), 138.89 (*ipso*-C of Ph), 138.99 (2 × *ipso*-C of Ph), 163.88 (d, $^2J_{CP}$ 3.1 Hz, C=O), ^{31}P NMR ($CDCl_3$, 162 MHz, ^{1}H-decoupled) δ 22.67 (P-5); HRMS (*m/z*) $[M + H]^+$ calcd. for $C_{47}H_{54}NO_9P$, 808.3609; found 808.3591.

5-deoxy-5-(diethylphosphonoacetamido)-*myo*-inositol (9a). To a solution of **8a** (107 mg, 0.132 mmol) in methanol (5 mL), THF (5 mL), deionised water (1 mL) and acetic acid (1 mL) was added palladium hydroxide on activated charcoal (20%, 50% water, 50 mg). The suspension was shaken in a Parr hydrogenator under H_2 (50 p.s.i.) for 72 h. The catalyst was removed by filtration through a PTFE syringe filter and the resulting colourless solution was concentrated, then dried under vacuum to give the title compound as a white solid (38 mg, 0.106 mmol, 80%); TLC (dichloromethane/methanol 3:1): R_f 0.06; ^{1}H NMR (400 MHz, D_2O) δ 1.31 (6 H, t, J 7.1 Hz, $POCH_2CH_3$), 3.05–3.12 (0.2 H, m, CH_2P partially deuterated), 3.56–3.70 (5 H, m, H-1, H-3, H-4, H-5 and H-6), 4.06 (1 H, t, J 2.6 Hz, H-2), 4.17 (4 H, apparent pentet, J 7.2 Hz, $POCH_2CH_3$); ^{13}C NMR (101 MHz, D_2O) δ 15.50 ($^3J_{CP}$ 6.0 Hz, $POCH_2CH_3$), 56.04 (C-5), 64.15 ($^2J_{CP}$ 6.5 Hz, $POCH_2CH_3$), 70.51 (2 × inositol ring *C*H), 71.92 (C-2 and 2 × inositol ring CH), 167.12 (d, $^2J_{CP}$ 5.9 Hz, C=O); ^{31}P NMR (162 MHz, D_2O, ^{1}H-

decoupled) δ 24.47 (1 P, s, P-5); HRMS (*m*/*z*) $[M + Na]^+$ calcd. for $C_{12}H_{22}D_2NO_9P$ (deuterated), 382.1206; found 382.1192.

5-deoxy-5-(diethylphosphonoacetamido)-*myo*-inositol 1,2,3,4,6-*O*-pentakis(dibenzylphosphate) (10a). To a stirred suspension of pentaol **9a** (38 mg, 0.106 mmol) and 5-phenyl-1*H*-tetrazole (116 mg 0.794 mmol) in dry dichloromethane (3 mL) under N_2 at room temperature was added bis(benzyloxy)-diisopropylaminophosphine (0.25 mL, 0.72 mmol). The mixture was stirred under N_2 at room temperature for 3 h and then cooled to –78 ºC, before *m*CPBA (57%, 320 mg, 1.06 mmol) was added. The mixture was allowed to warm to room temperature and then diluted with EtOAc (30 mL). The clear, colourless solution was washed with 10% w/v aq. Na_2SO_3 solution (2 × 35 mL), dried over $MgSO_4$ and concentrated. The residue was purified by flash chromatography (MeOH in dichloromethane, 0 to 5%) to give **10a** as a colourless oil (117 mg, 0.071 mmole, 67%); TLC (dichloromethane:MeOH, 30:1): R_f 0.30; ^{1}H NMR ($CDCl_3$, 400 MHz) δ 1.19 (6 H, t, *J* 7.1 Hz, $POCH_2CH_3$), 2.85–2.91 (~0.3 H, m, CH_2P partially deuterated), 3.98–4.12 (4 H, m, 2 × $POCH_2CH_3$), 4.23 (1 H, broad m, H-5), 4.39 (2 H, tt, *J* 9.4, 2.1 Hz, H-1 and H-3), 4.86–5.10 (20 H, m, $POCH_2Ph$, H-4 and H-6), 5.15–5.19 (2 H, $POCH_2Ph$), 5.65 (1 H, dt, *J* 9.1, 2.4 Hz, H-2), 6.87(1 H, d, *J* 9.1 Hz, NH); 7.14–7.28 (50 H, m, Ph); ^{13}C NMR (101 MHz, $CDCl_3$) δ 16.28 (d, $^3J_{CP}$ 6.1 Hz, $POCH_2CH_3$), 53.04 (broad, C-5), 62.28 (d, $^2J_{CP}$ 6.0 Hz, $POCH_2CH_3$), 69.67–70.05 (overlapping signals with J_{CP} couplings, $POCH_2Ph$), 74.49 (with J_{CP} couplings, C-1 and C-3), 74.68 (with J_{CP} couplings, C-4 and C-6), 76.21 (with J_{CP} couplings, C-2), 127.85, 127.89, 128.08, 128.12, 128.16, 128.24, 128.37, 128.44, 128.47 and 128.50 (*C*H of Ph), 135.58–135.79 (overlapping signals with J_{CP} couplings, *ipso*-C of Ph), 166.31 ($^2J_{CP}$ 3.1 Hz, C=0); ^{31}P NMR (162 MHz, $CDCl_3$, ^{1}H-decoupled) δ –2.35 (1 P, P-2), –1.68 (2 P), –0.81 (2 P), 21.57 (1 P, P-5).

5-deoxy-5-(phosphonoacetamido)-*myo*-inositol 1,2,3,4,6-pentakisphosphate (1a). A stirred solution of **10a** (68 mg, 41 μmole) in dry dichloromethane (2 mL) was cooled to 0 °C under N_2 and trimethylsilyl bromide (1 mL) was added dropwise over 5 min. The solution was allowed to warm gradually to room temperature, and stirring was continued for 48 h. The solution was concentrated and methanol (5 mL) was added to the residue. The resulting colourless solution was stirred at room temperature for a further 1 h, then concentrated to give a white gum. The gum was washed with diethyl ether (3 × 2 mL), then taken up in aqueous TEAB (1.0 moldm^{-3}, pH 7.6, 5 mL). The solution was then washed with diethyl ether (3 x 5 mL) and concentrated. The residue was re-dissolved in MilliQ water and lyophilised to give the triethylammonium salt of the title compound **11** as a white powder (46 mg, 35 μmole, 85 %); ^{1}H NMR (500 MHz,

D_2O) δ 1.27 (~56 H, t, J 7.3 Hz, CH_3 of TEA^+), 2.77–2.82 (~0.3 H, m, CH_2P, partially deuterated), 3.18 (~34 H, q, J 7.3 Hz, CH_2 of TEA^+), 4.06 (1 H, broad t, J 9.6 Hz, H-5), 4.28 (2 H, t, J = 9.5 Hz, H-1 and H-3), 4.45 (2 H, q, J 9.8 Hz, H-4 and H-6), 4.85 (1 H, d, J 10.0 Hz, H-2), ^{13}C NMR (100 MHz, D_2O) δ 8.21 (CH_3 of TEA^+), 46.60 (CH_2 of TEA^+), 53.80 (C-5), 74.42 (with J_{CP} couplings, C-1 and C-3), 74.89 (with J_{CP} couplings, C-4 and C-6), 76.03 (with J_{CP} couplings, C2), 170.56 (d, $^2J_{CP}$ approx. 6 Hz, C=O); ^{31}P NMR (162 MHz, D_2O, ^1H-decoupled) δ 0.24 (1 P, P-2), 0.72 (2 P), 1.03 (2 P), 14.11 (1 P, P-5); HRMS (*m*/*z*) [M – H]$^-$ calcd. for $C_8H_{19}D_2NO_{24}P_6$ (deuterated), 701.8932; found 701.8972.

NMR Spectra

Compound **1**; ^{1}H NMR (400 MHz, D_2O)

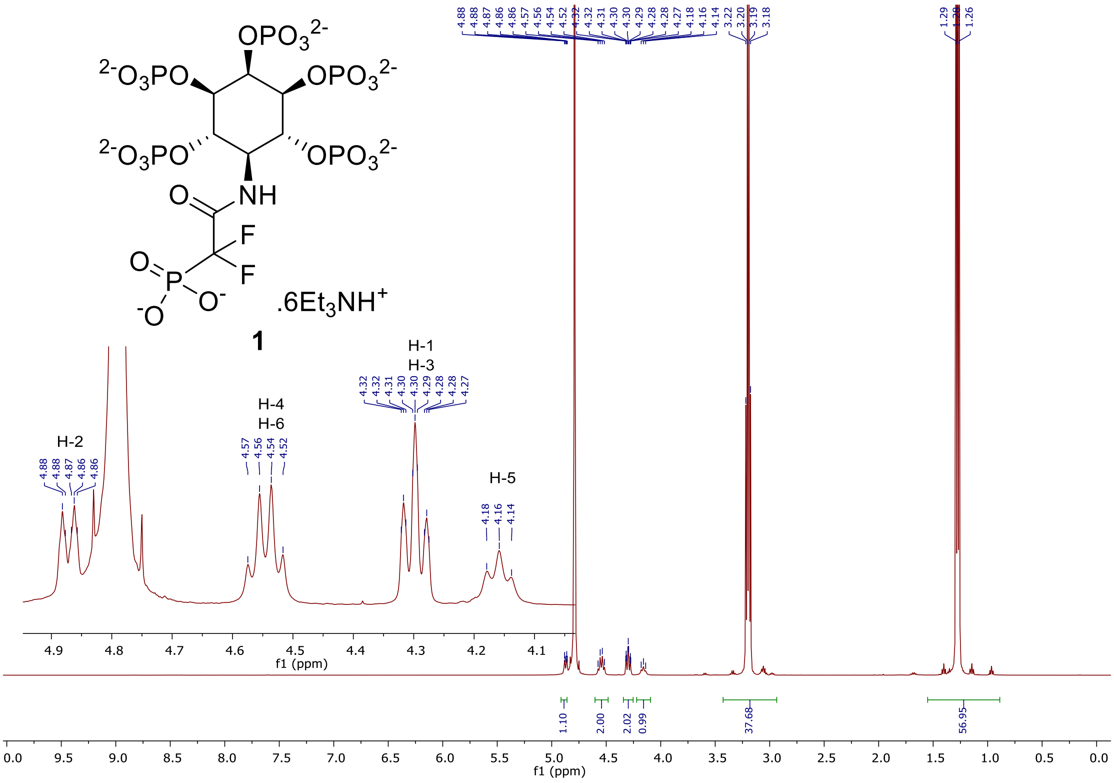

Compound **1**; ^{13}C NMR (126 MHz, D_2O)

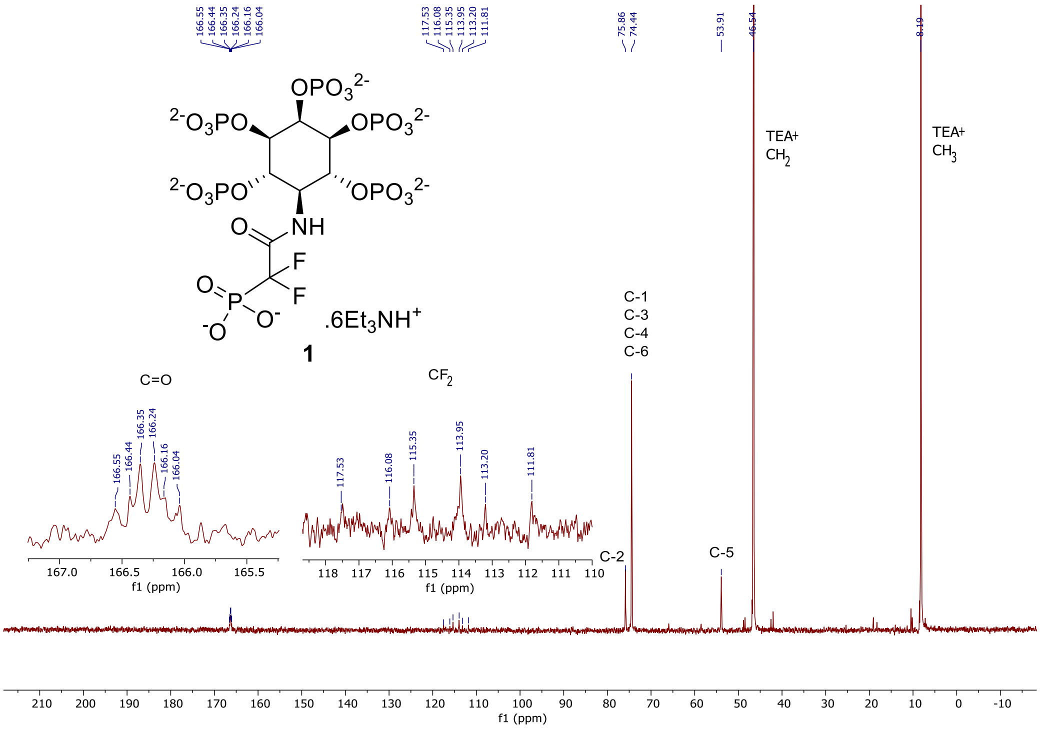

Compound **1**; ^{31}P NMR (126 MHz, D_2O)

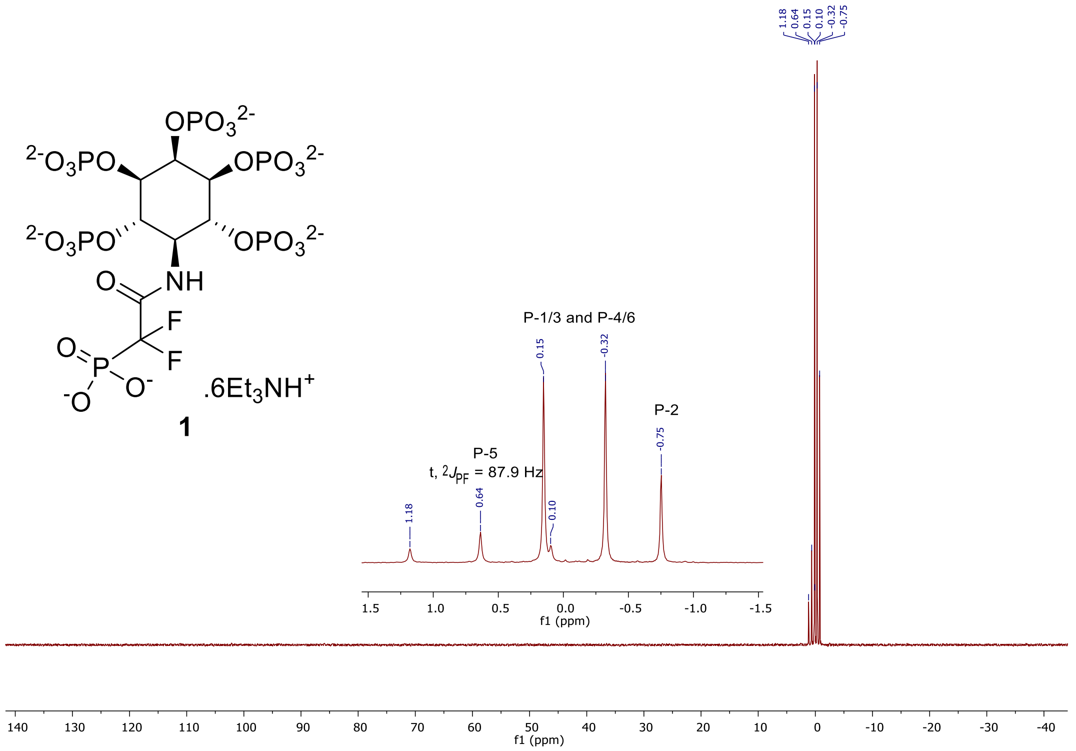

Compound **1**; ^{19}F NMR (471 MHz, D_2O)

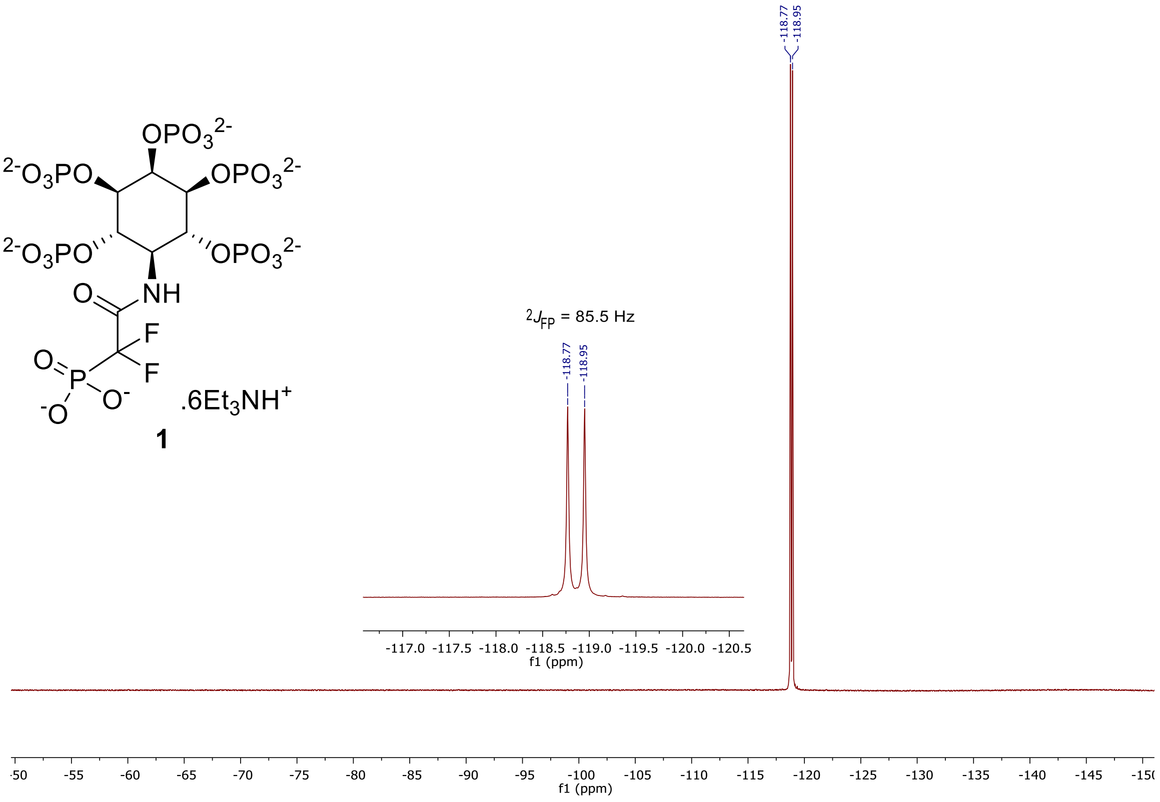

Compound **1a**; ^{1}H NMR (500 MHz, D_2O)

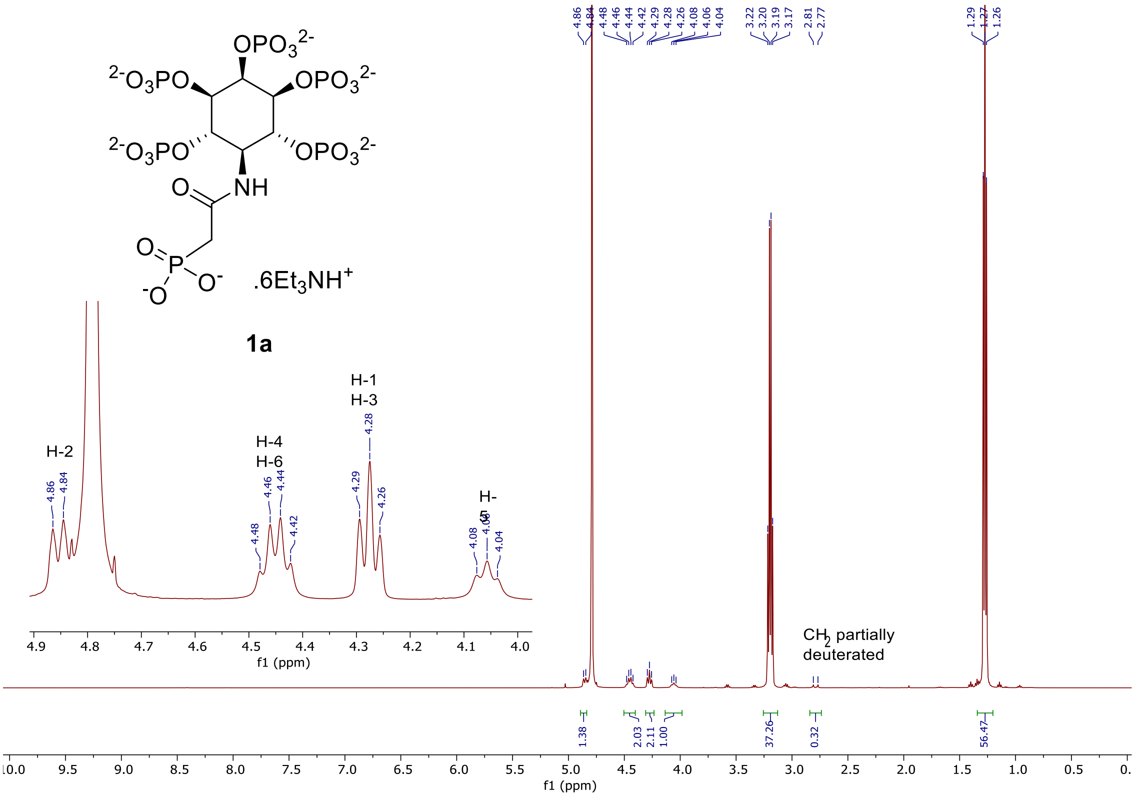

Compound **1a**; ^{13}C NMR (100 MHz, D_2O)

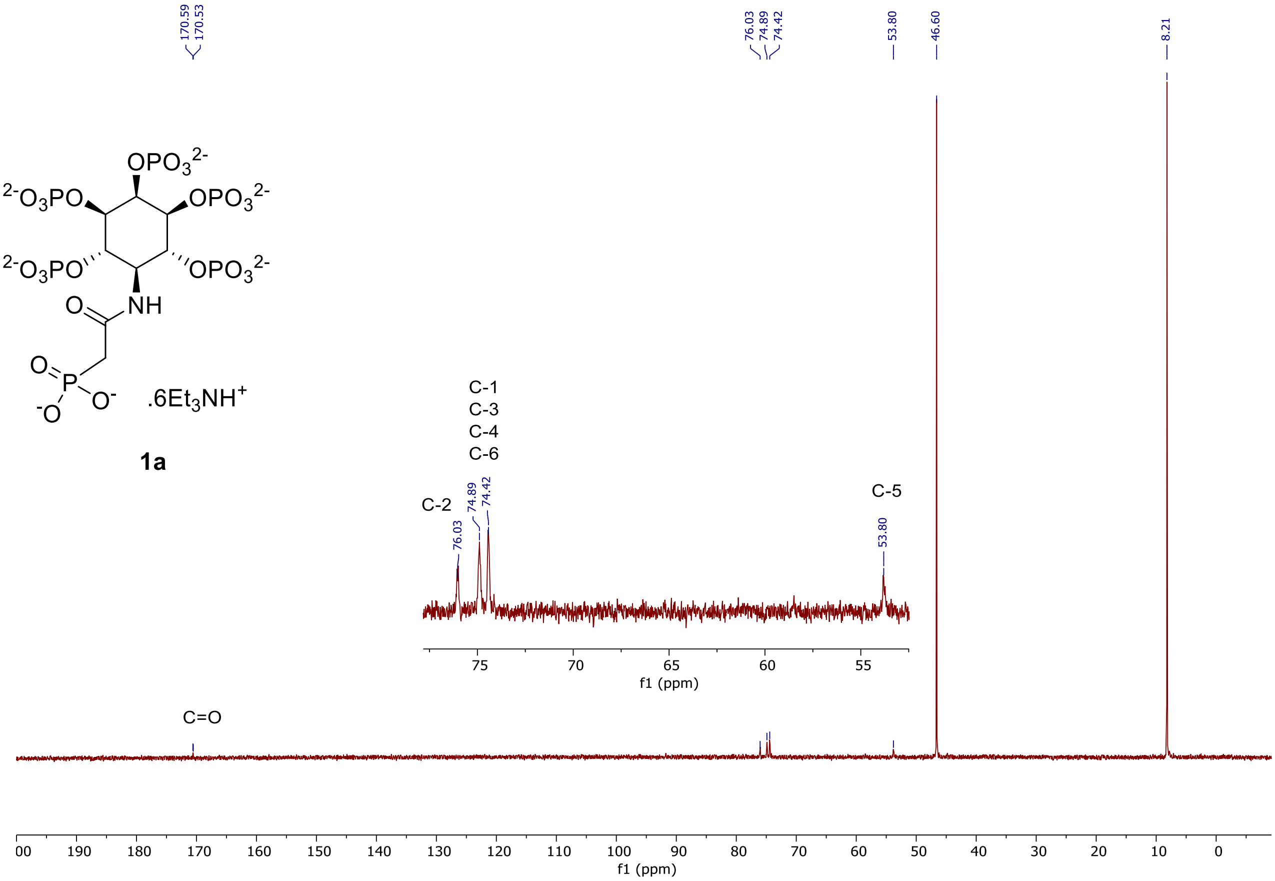

Compound **1a**; ^{31}P NMR (162 MHz, D_2O, ^{1}H-decoupled)

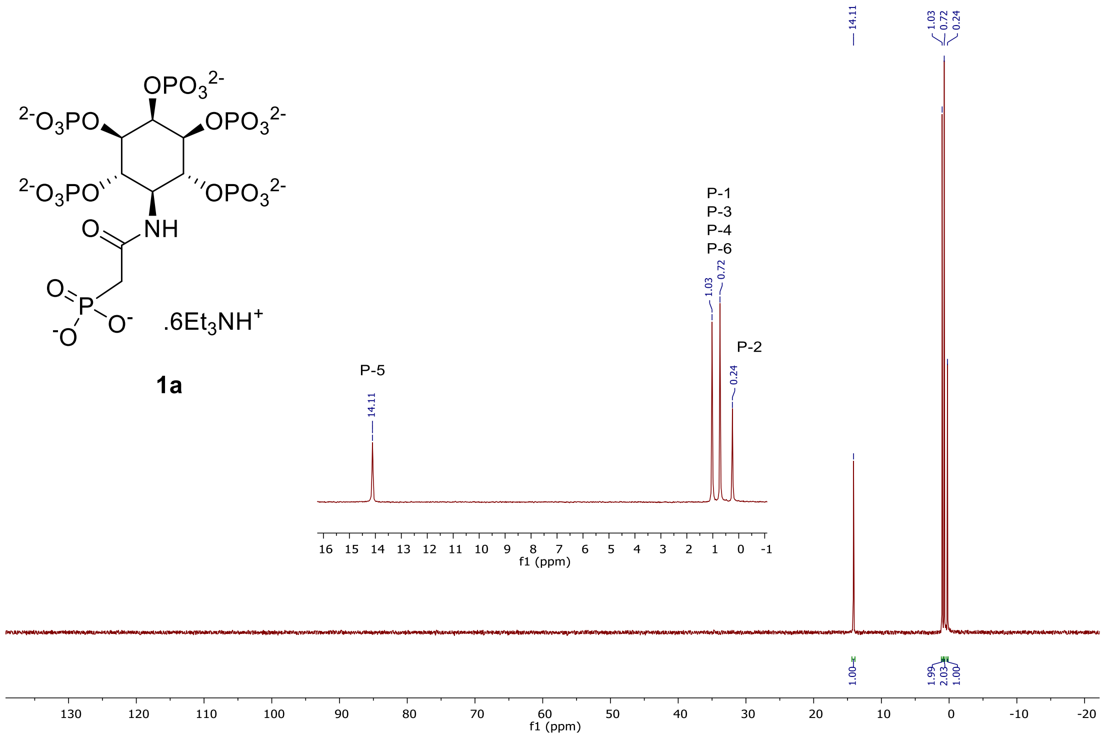

Compound **4**; ^{1}H NMR (400 MHz, $CDCl_3$)

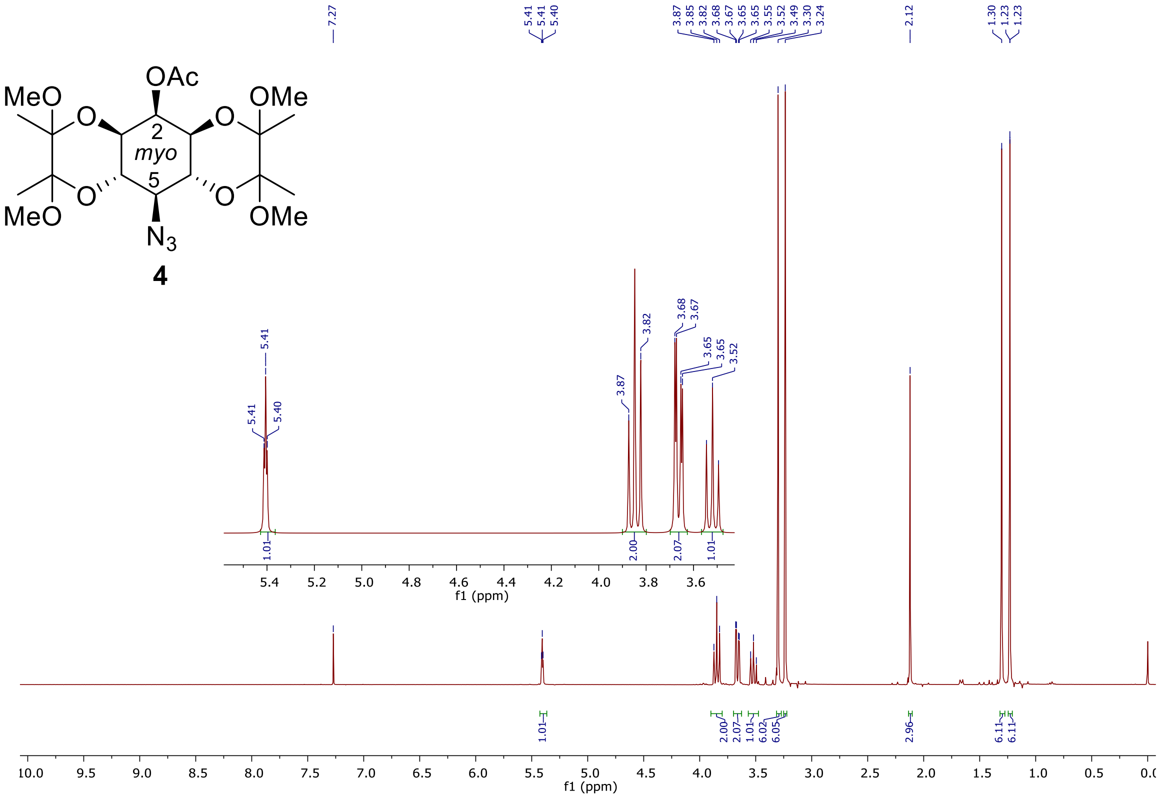

Compound **4**; ^{13}C NMR (100 MHz, CDCl$_3$

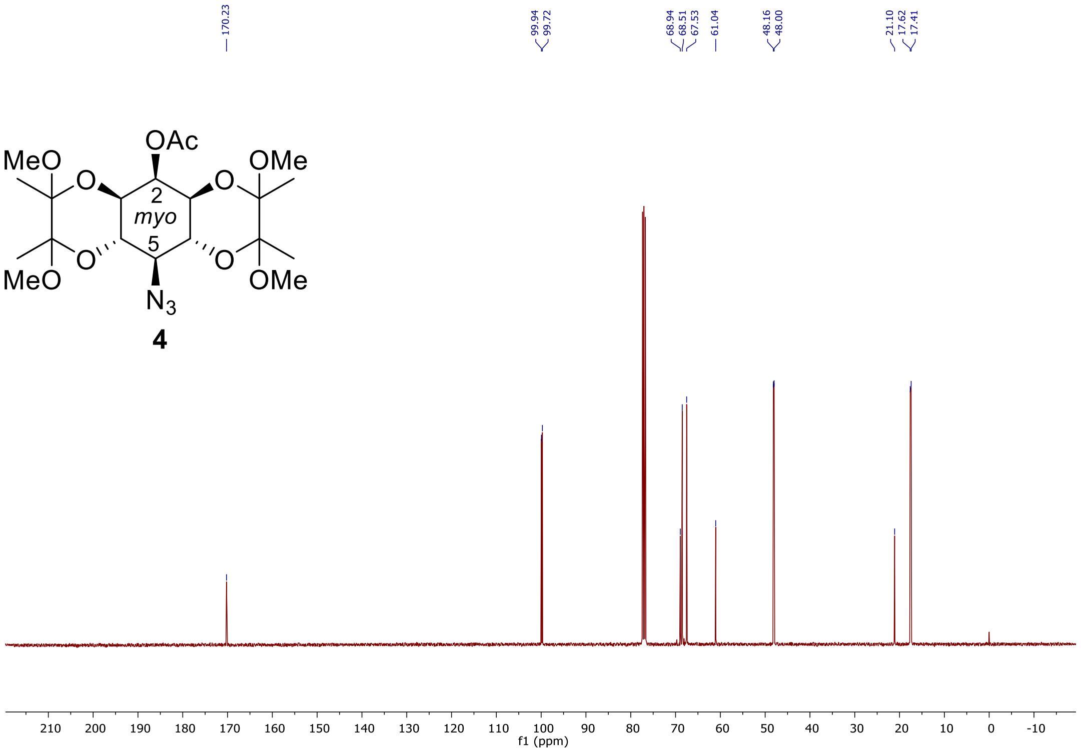

Compound **5**; ^{1}H NMR (400 MHz, CDCl$_3$)

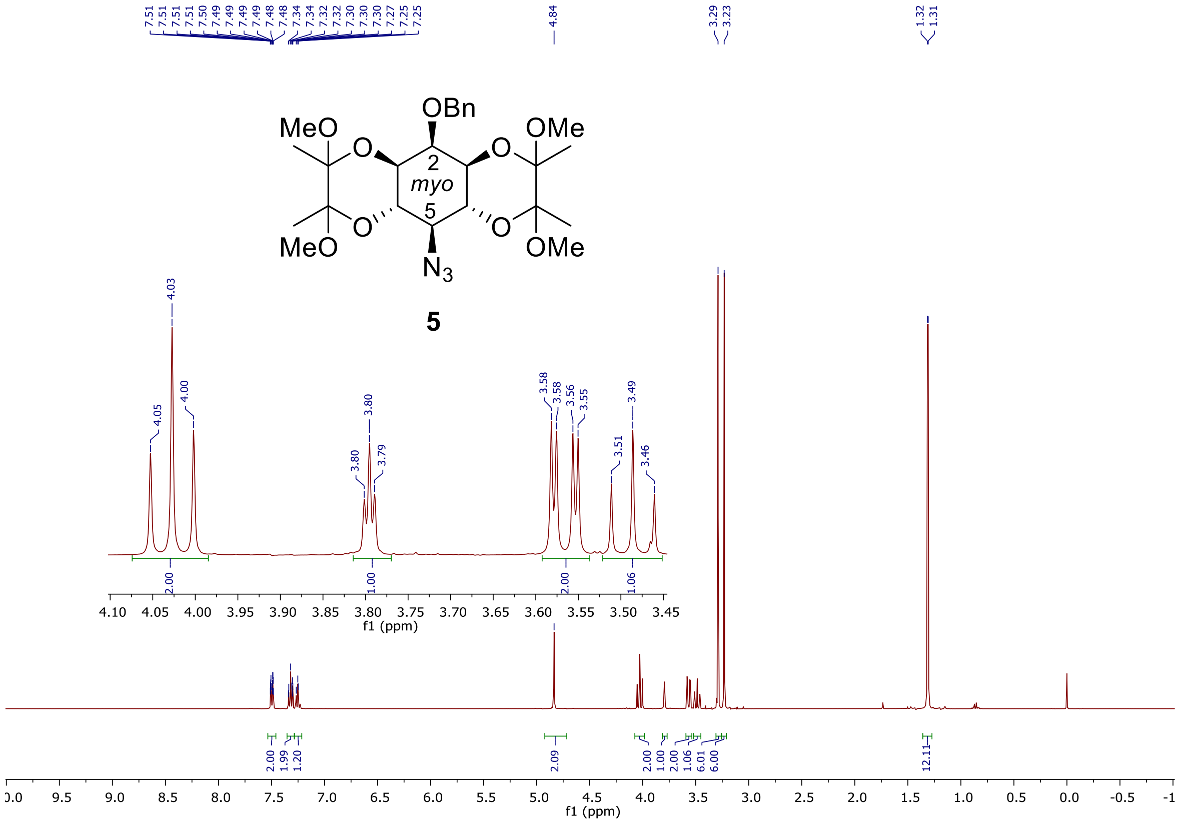

Compound **5**; ^{13}C NMR (100 MHz, CDCl$_3$)

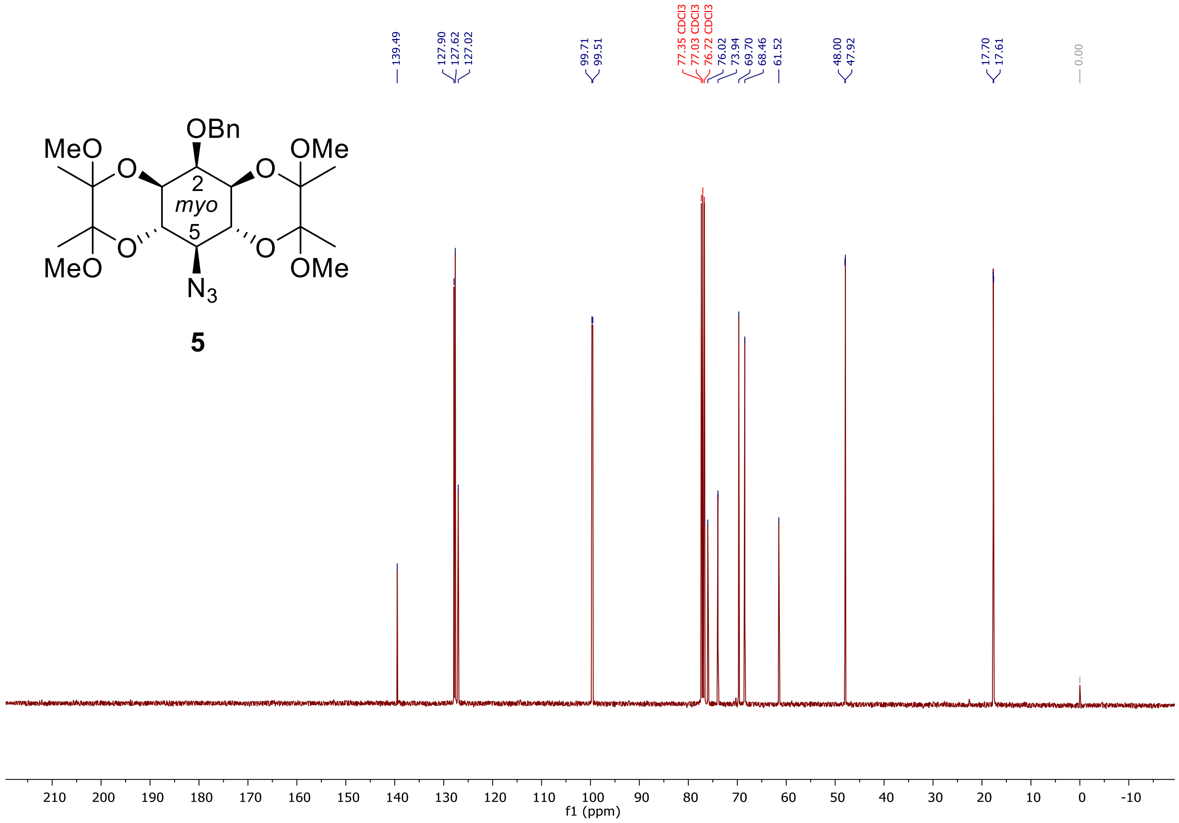

Compound **6**; ^{1}H NMR (400 MHz, CDCl$_3$)

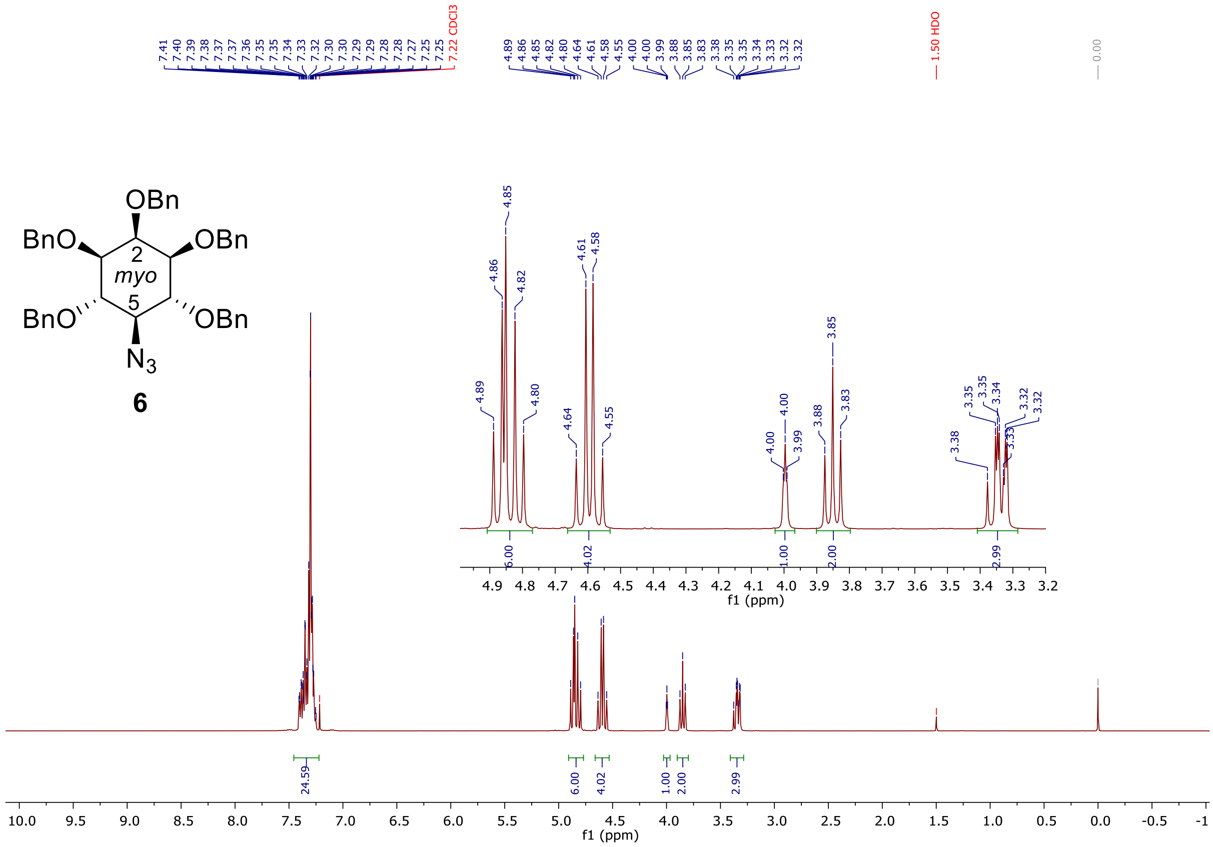

Compound **6**; ^{13}C NMR (100 MHz, $CDCl_3$)

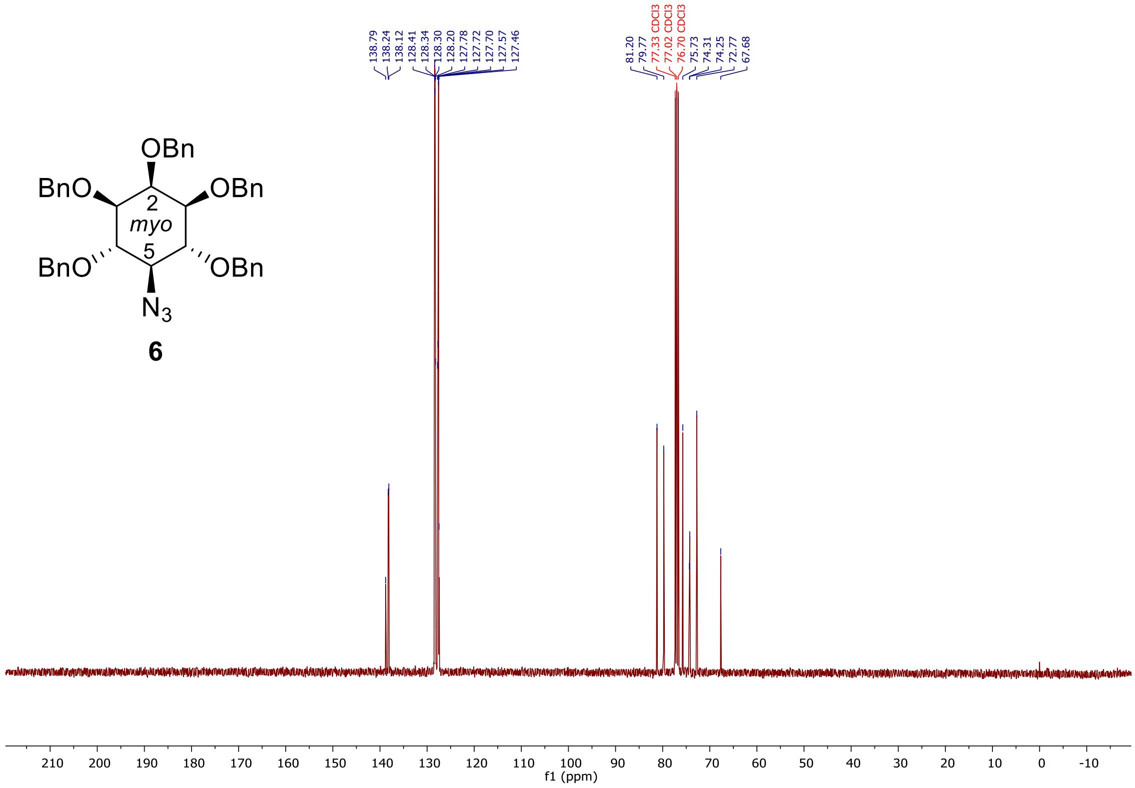

Compound **7**; ^{1}H NMR (400 MHz, $CDCl_3$)

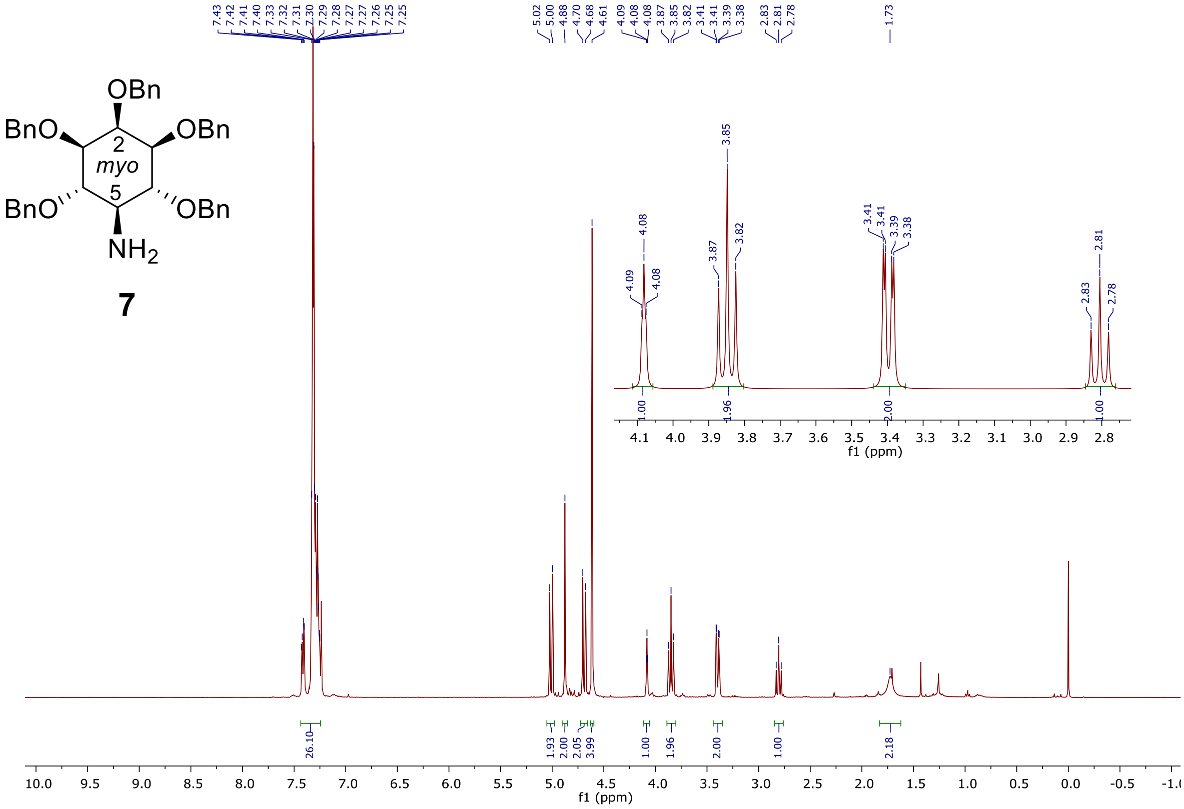

Compound **7**; ^{13}C NMR (100 MHz, $CDCl_3$)

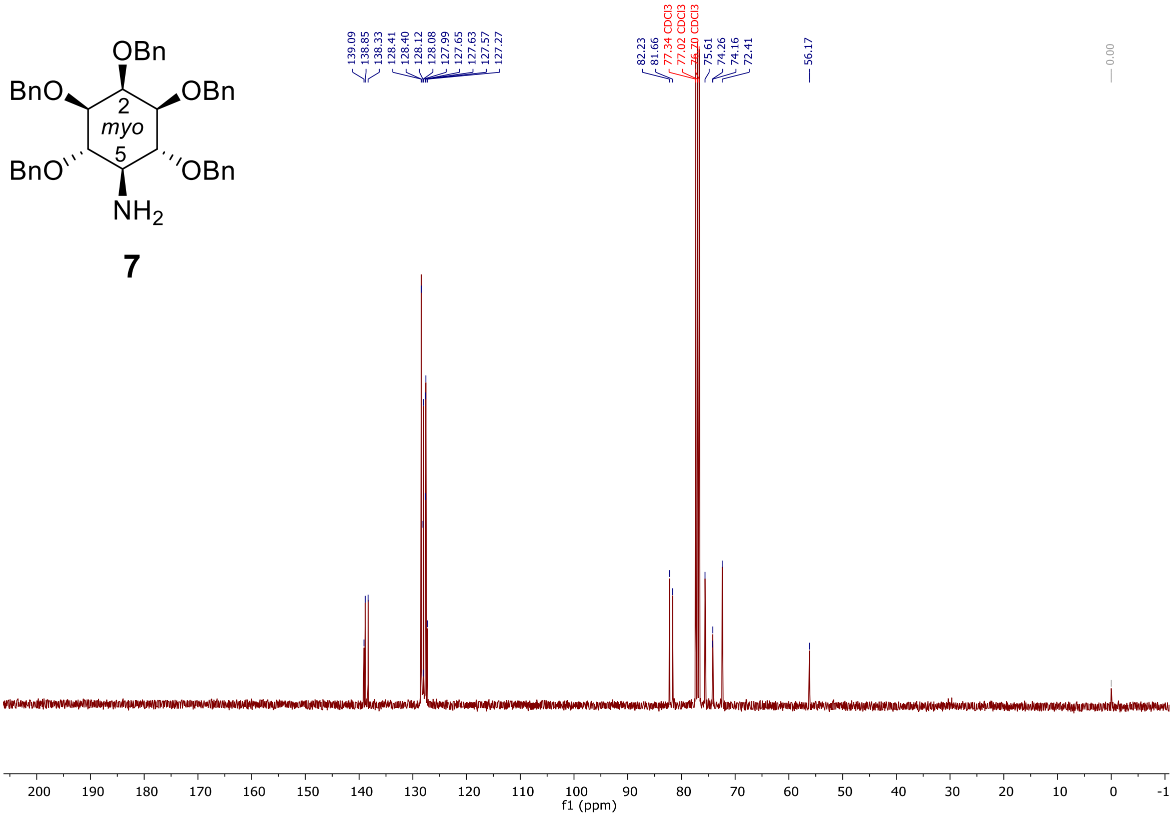

Compound **8**; ^{1}H NMR (400 MHz, $CDCl_3$)

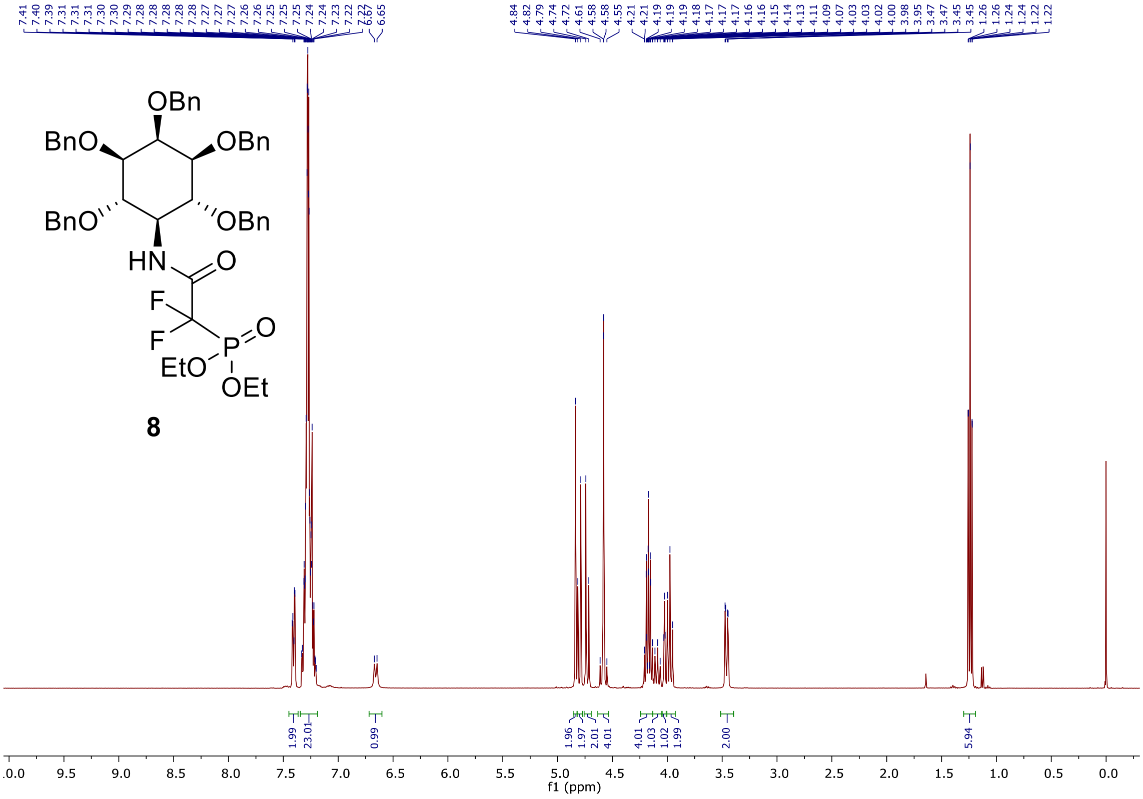

Compound **8**; ^{13}C NMR (100 MHz, $CDCl_3$)

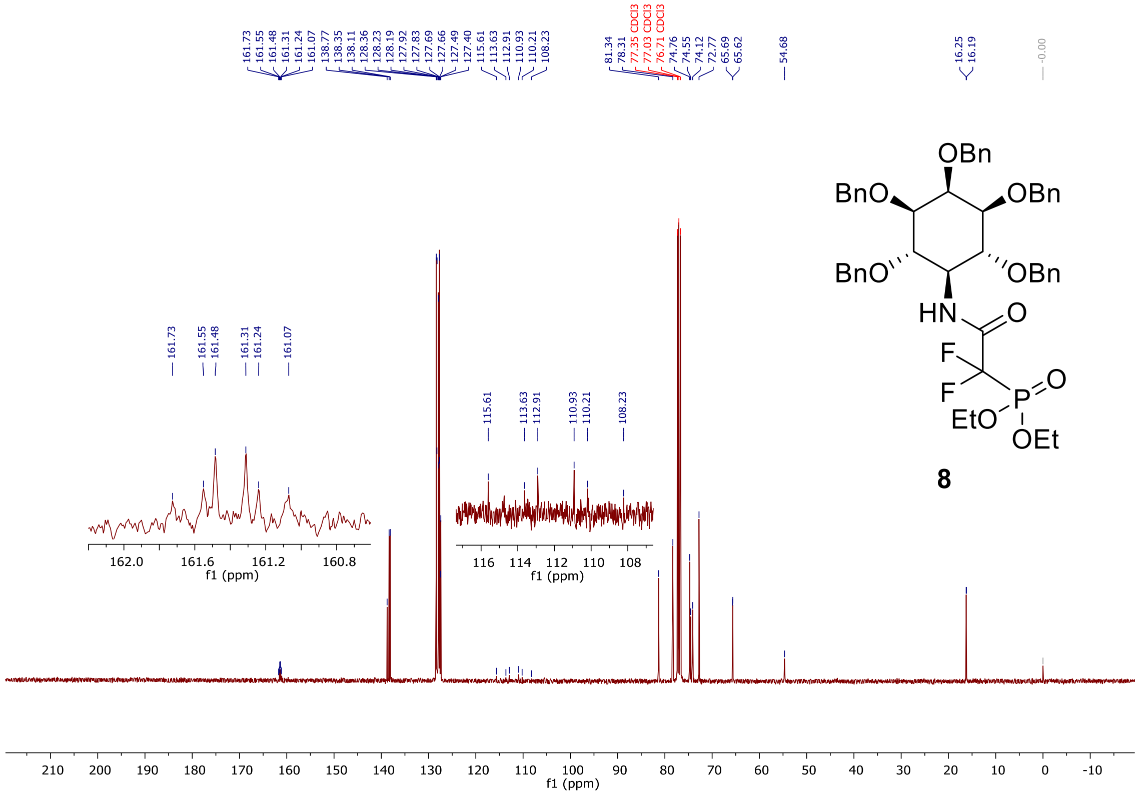

Compound **8**; ^{31}P NMR (162 MHz, $CDCl_3$, ^{1}H-decoupled)

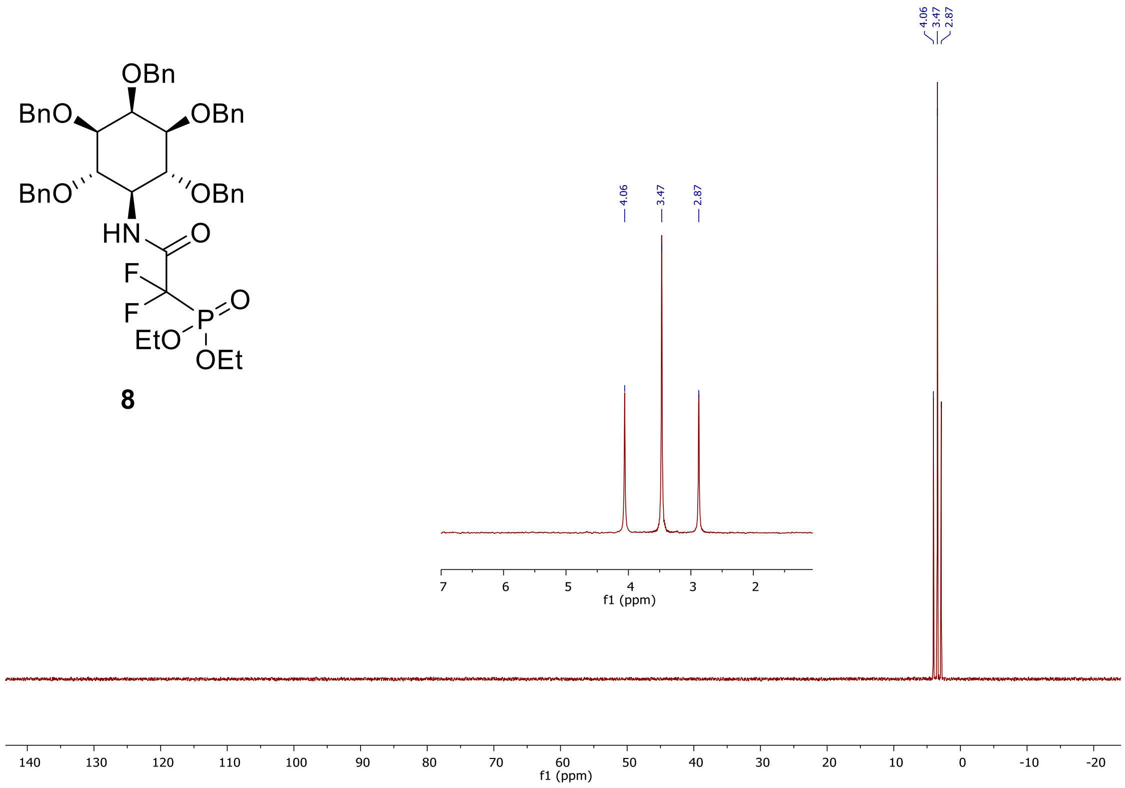

Compound **8**; ^{19}F NMR (471 MHz, CDCl$_3$)

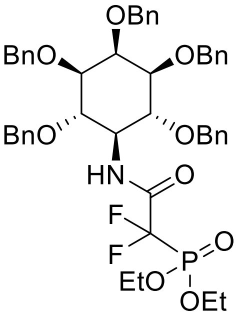

8

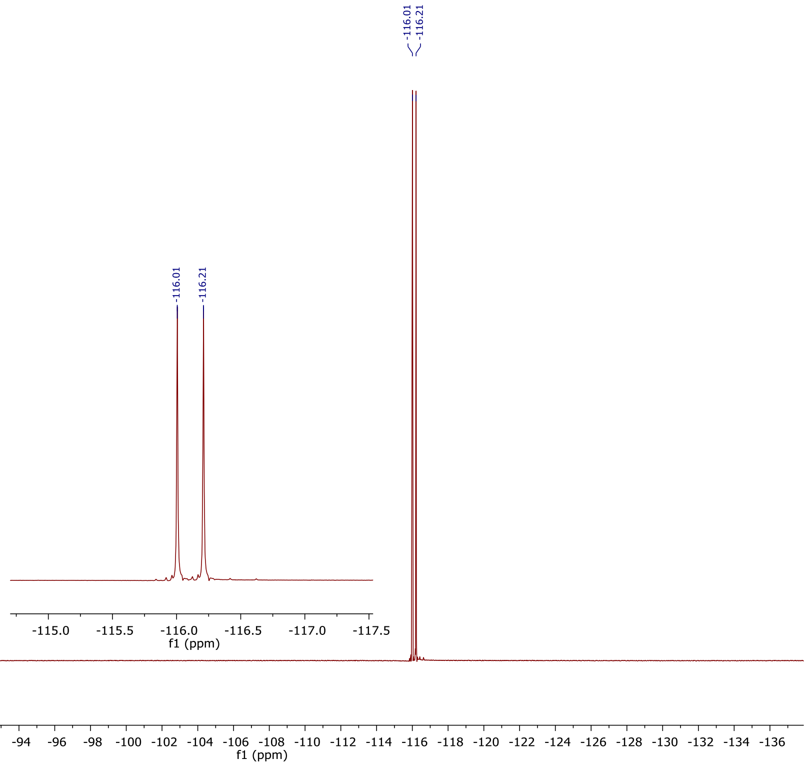

Compound **8a**; ^{1}H NMR (400 MHz, CDCl$_3$)

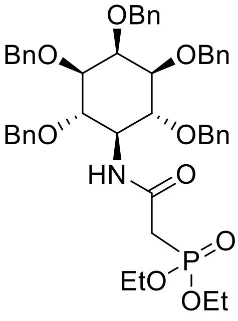

8a

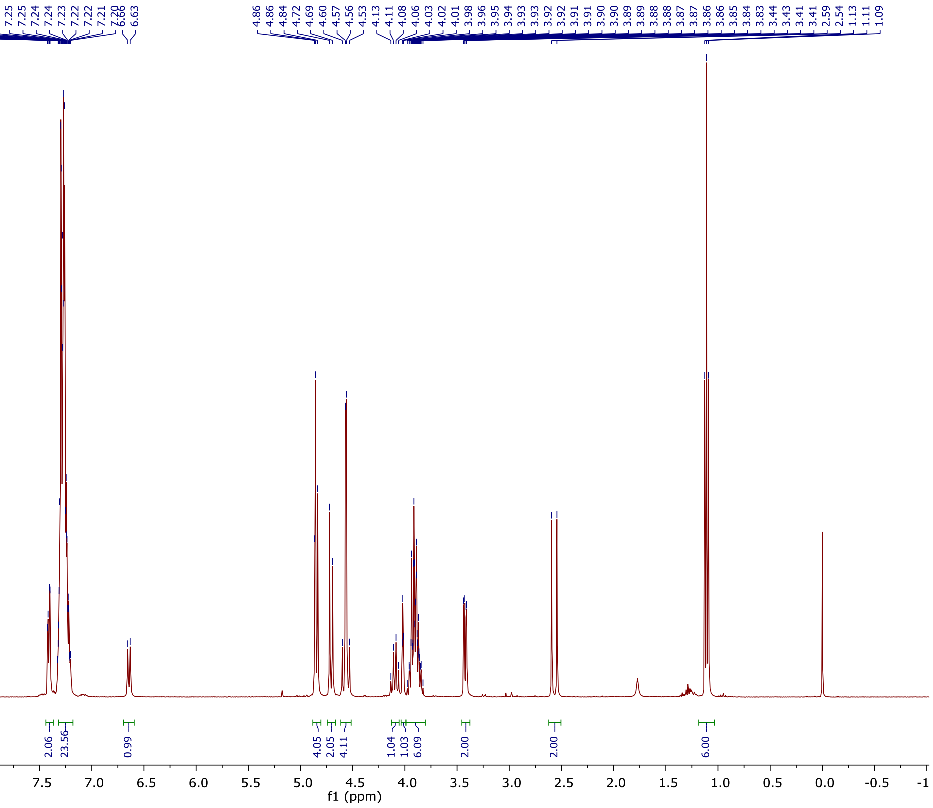

Compound **8a**; ^{13}C NMR (100 MHz, $CDCl_3$)

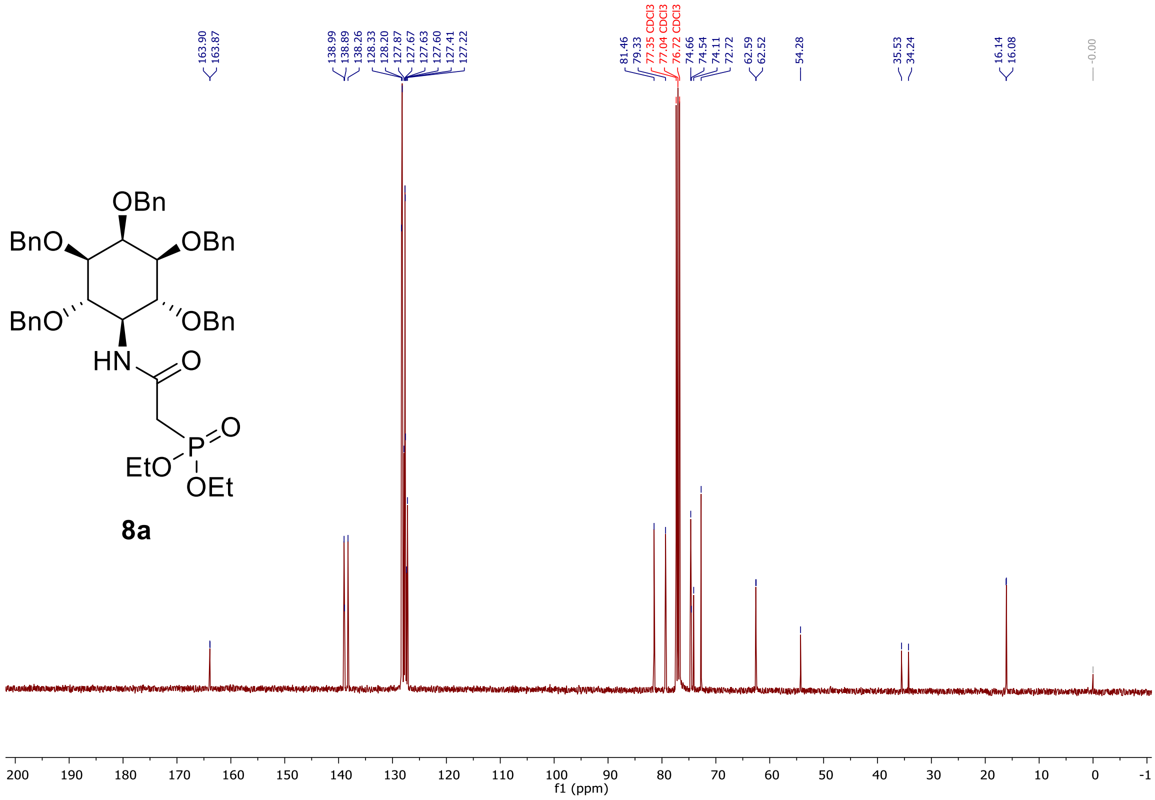

Compound **8a**; ^{31}P NMR (162 MHz, $CDCl_3$, ^{1}H-decoupled)

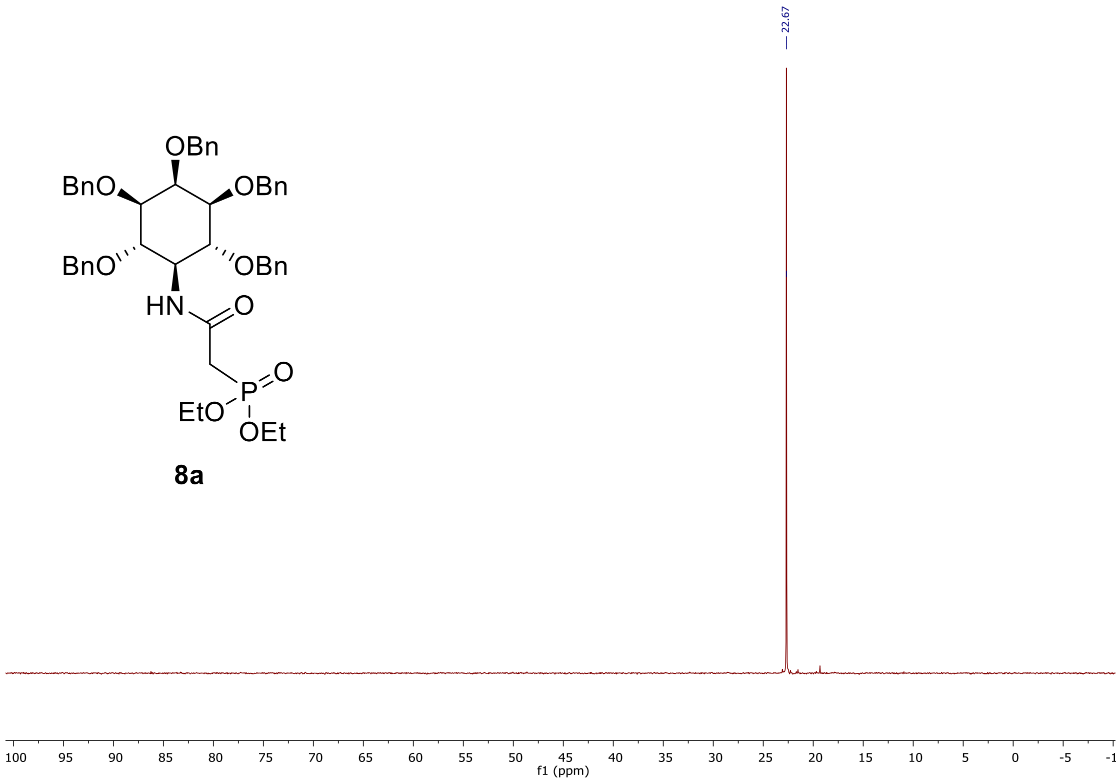

Compound **9**; ^{1}H NMR (400 MHz, D_2O)

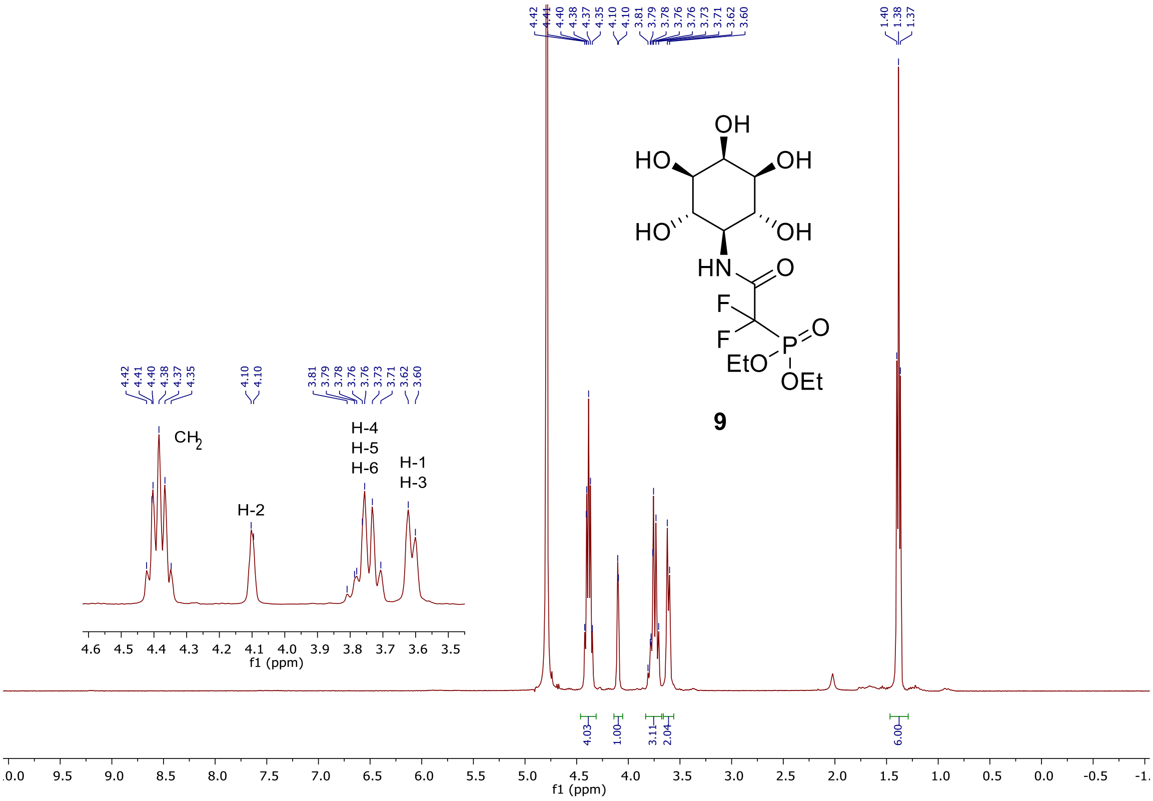

Compound **9**; ^{13}C NMR (100 MHz, D_2O)

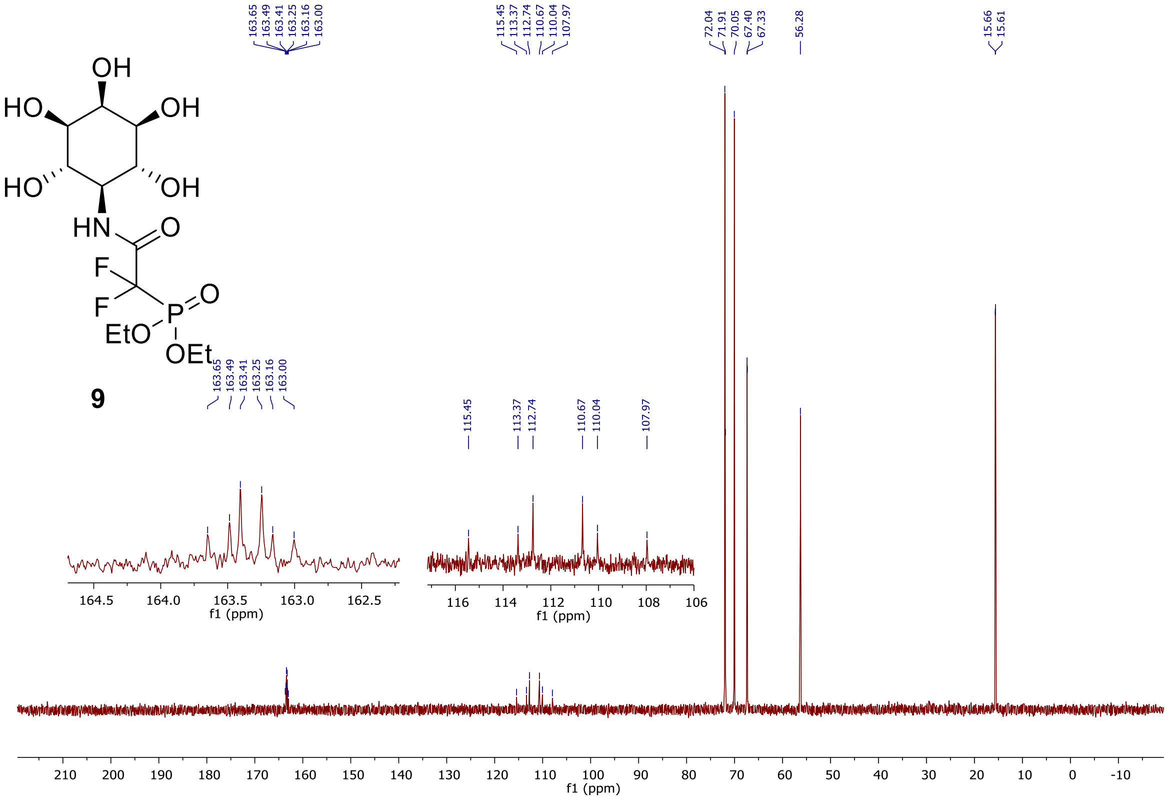

Compound **9**; ^{31}P NMR (162 MHz, D_2O, ^{1}H-decoupled)

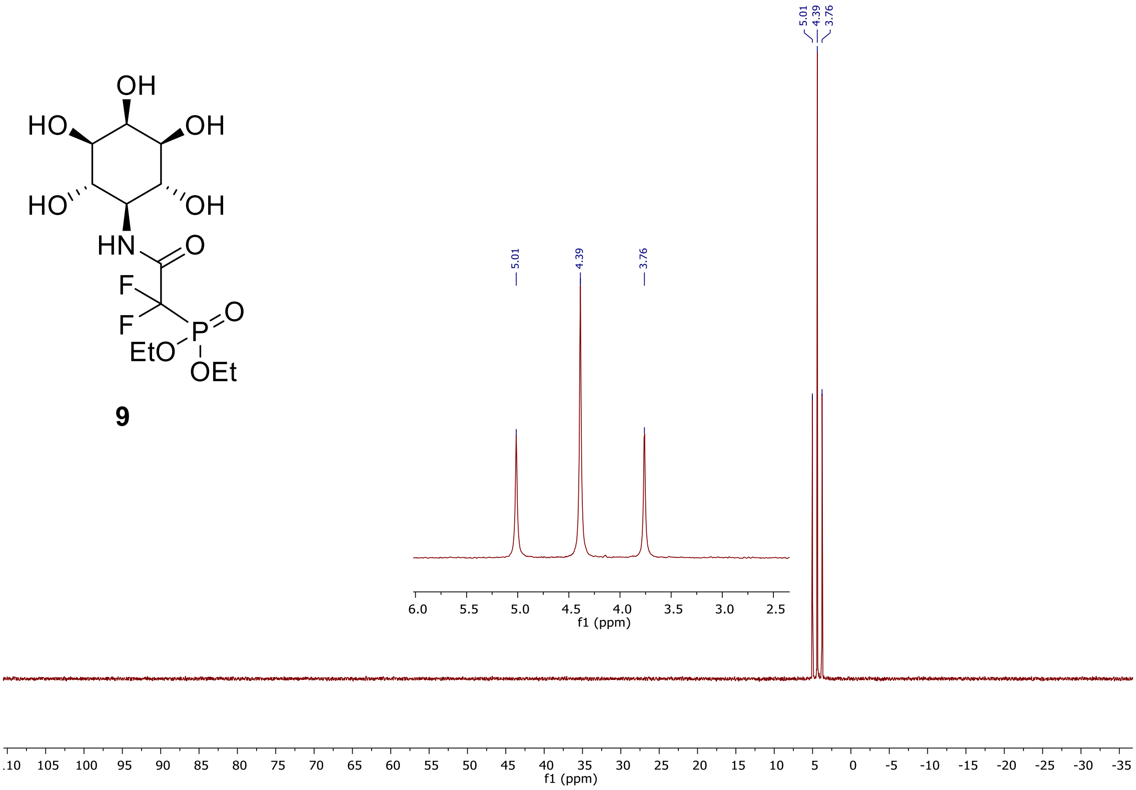

Compound **9**; ^{19}F NMR (471 MHz, D_2O)

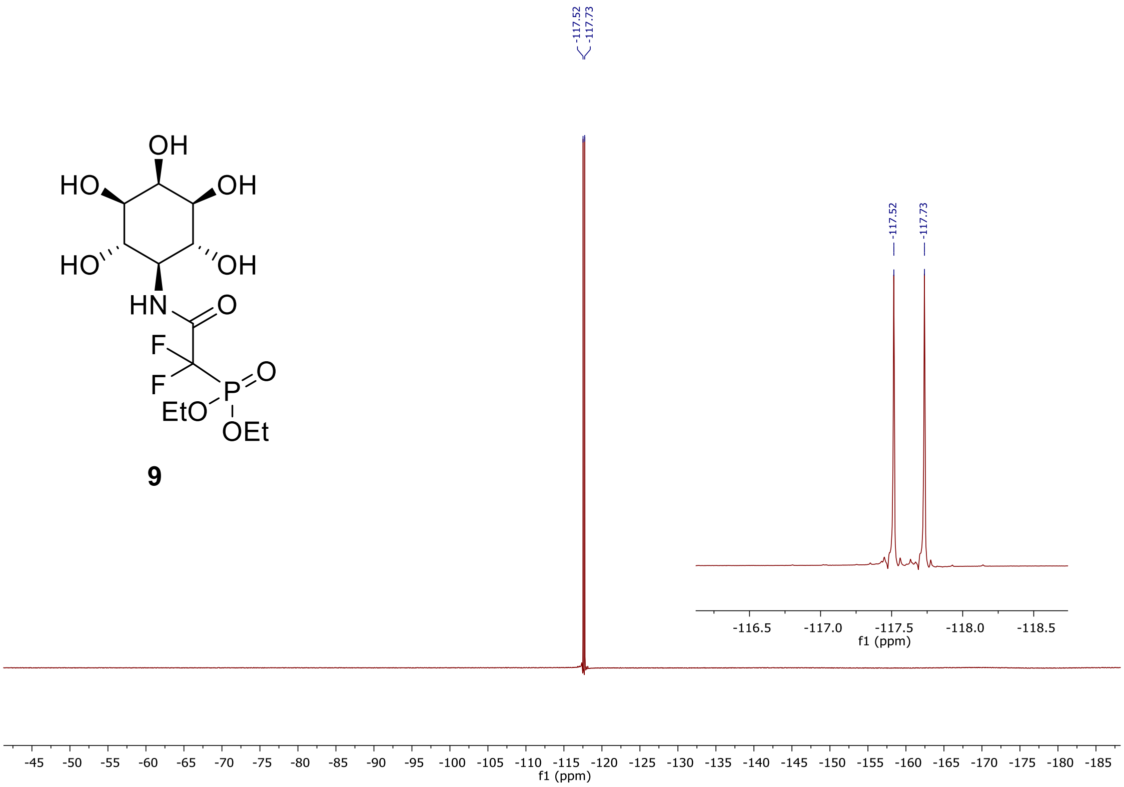

Compound **9a**; ^{1}H NMR (400 MHz, D_2O)

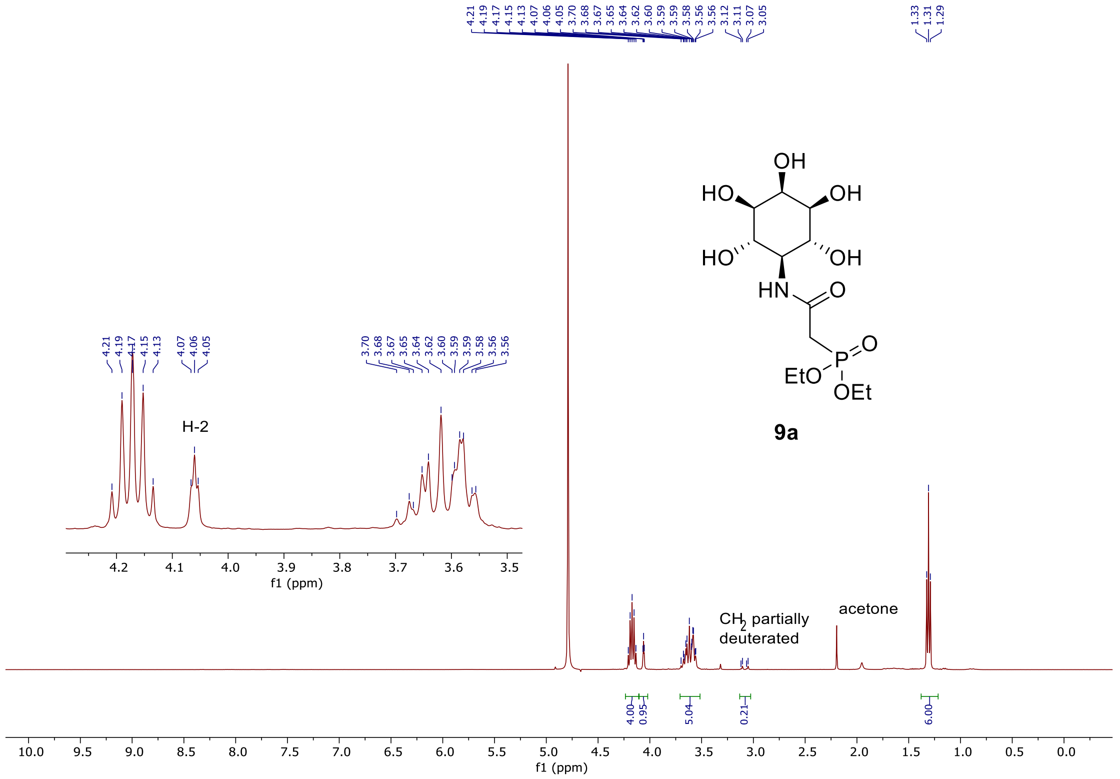

Compound **9a**; ^{13}C NMR (100 MHz, D_2O)

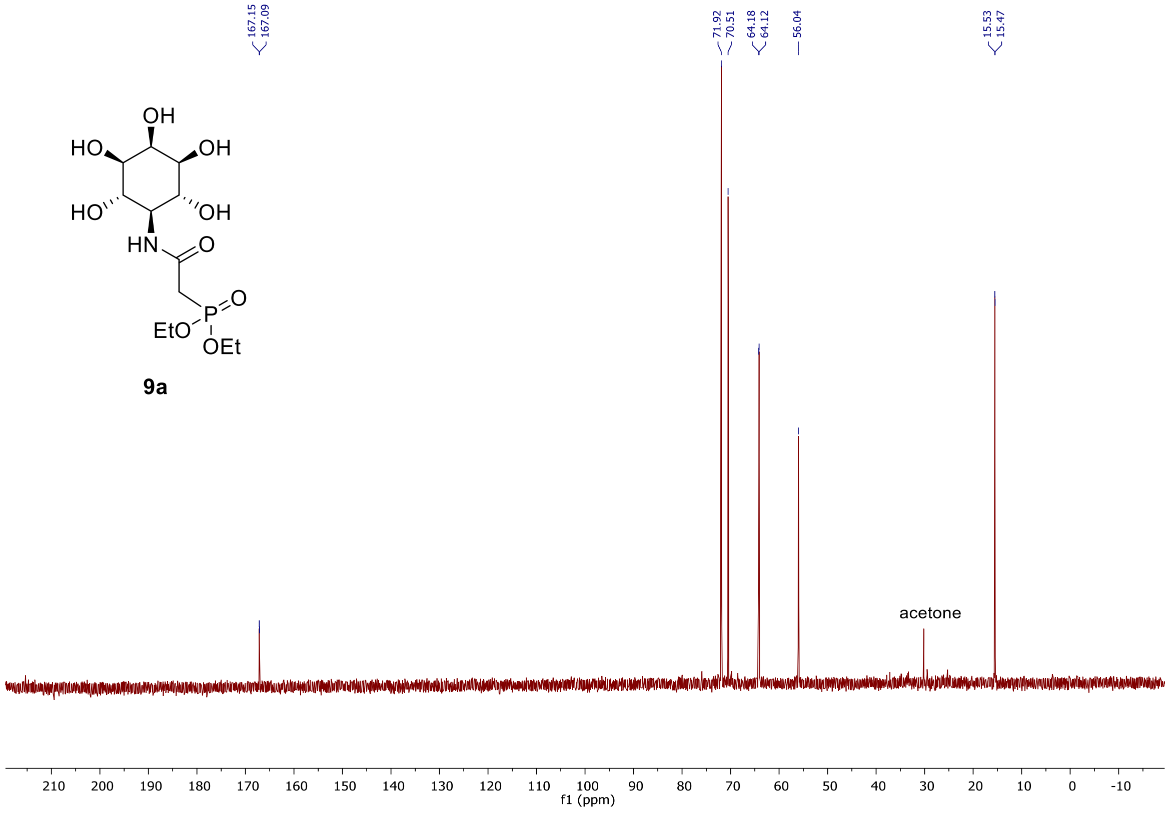

Compound **9a**; ^{31}P NMR (162 MHz, D_2O, ^{1}H-decoupled)

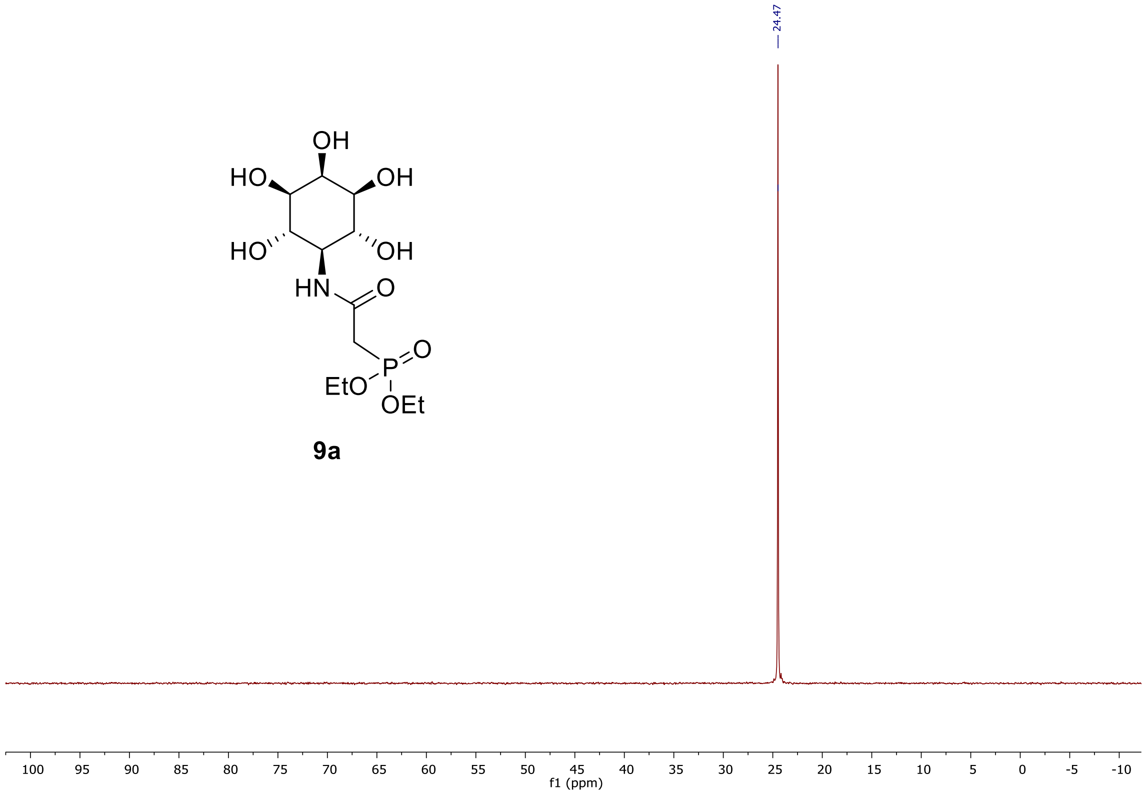

Compound **10**; ^{1}H NMR (400 MHz, $CDCl_3$)

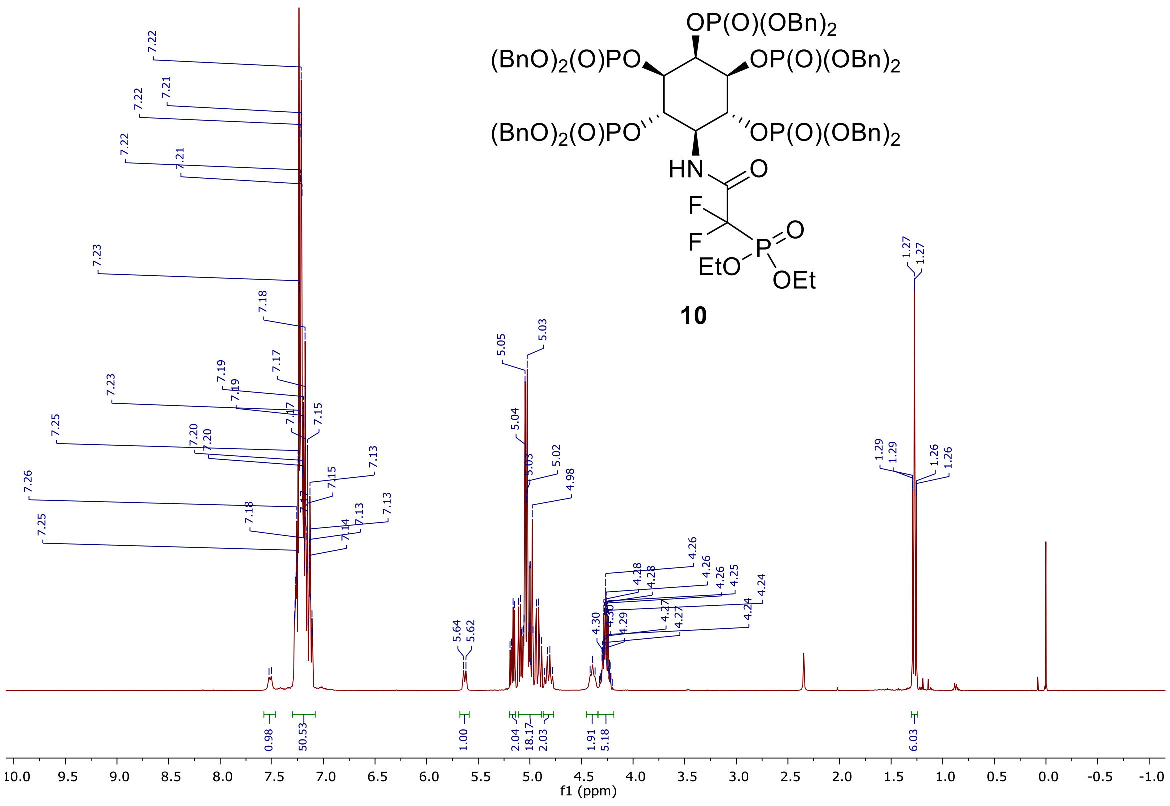

Compound **10**; ^{13}C NMR (100 MHz, $CDCl_3$)

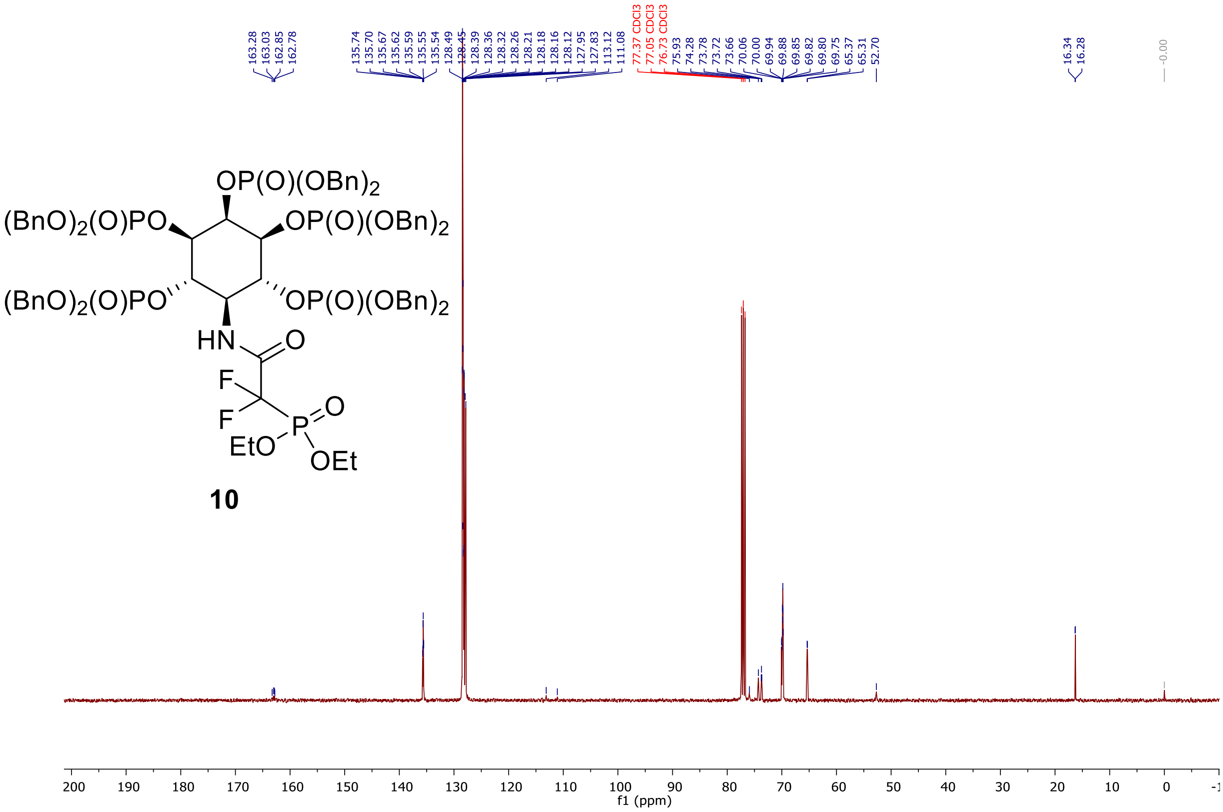

Compound **10**; ^{31}P NMR (162 MHz, $CDCl_3$, ^1H-decoupled)

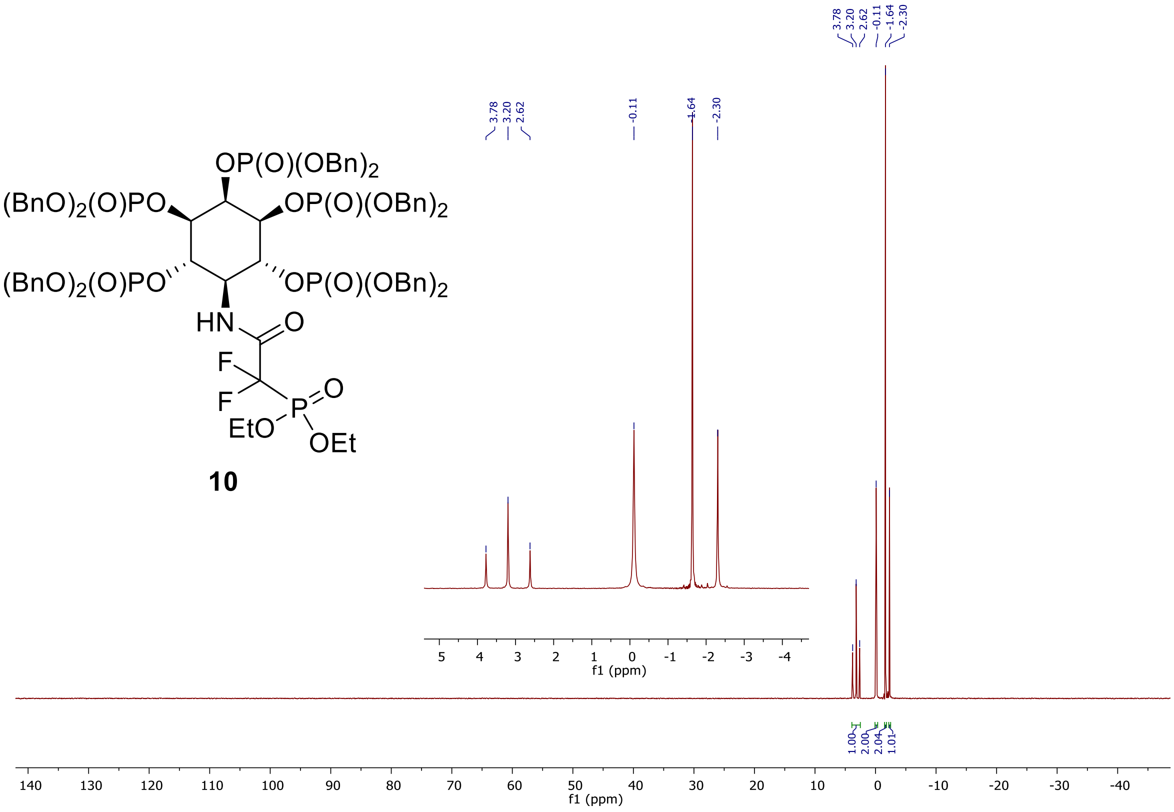

Compound **10**; ^{19}F NMR (471 MHz, $CDCl_3$)

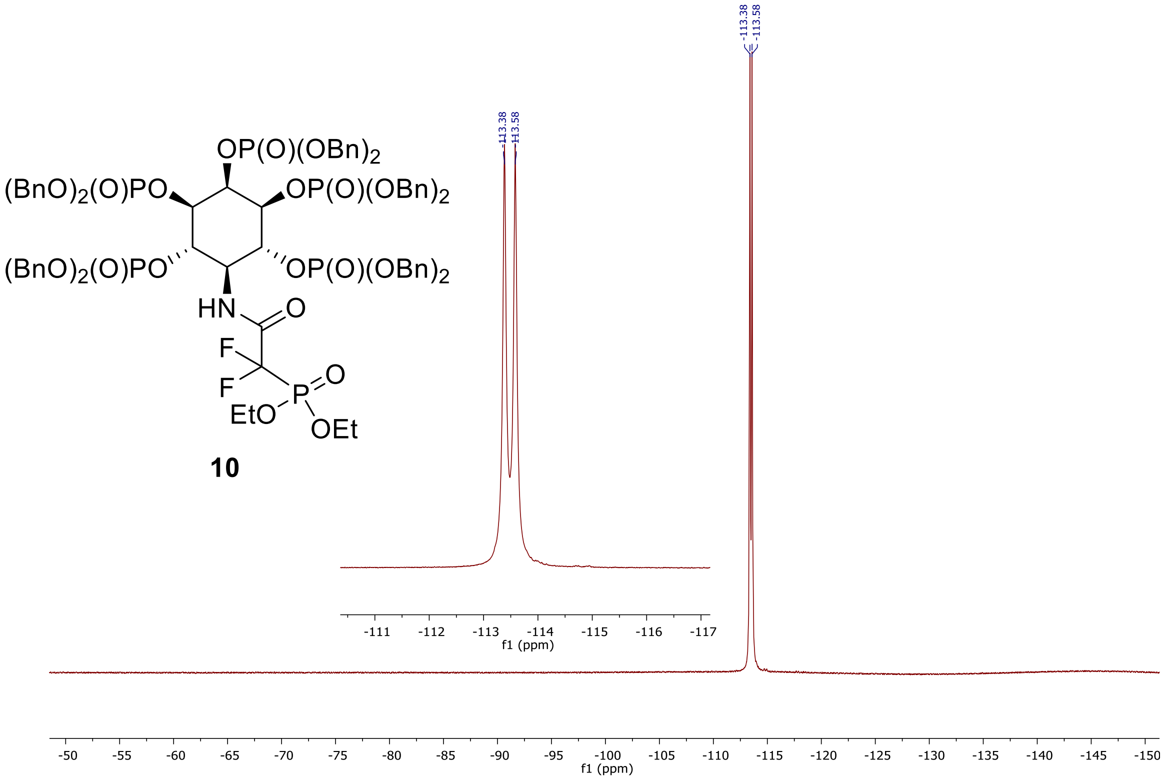

Compound **10a**; ^{1}H NMR (162 MHz, $CDCl_3$)

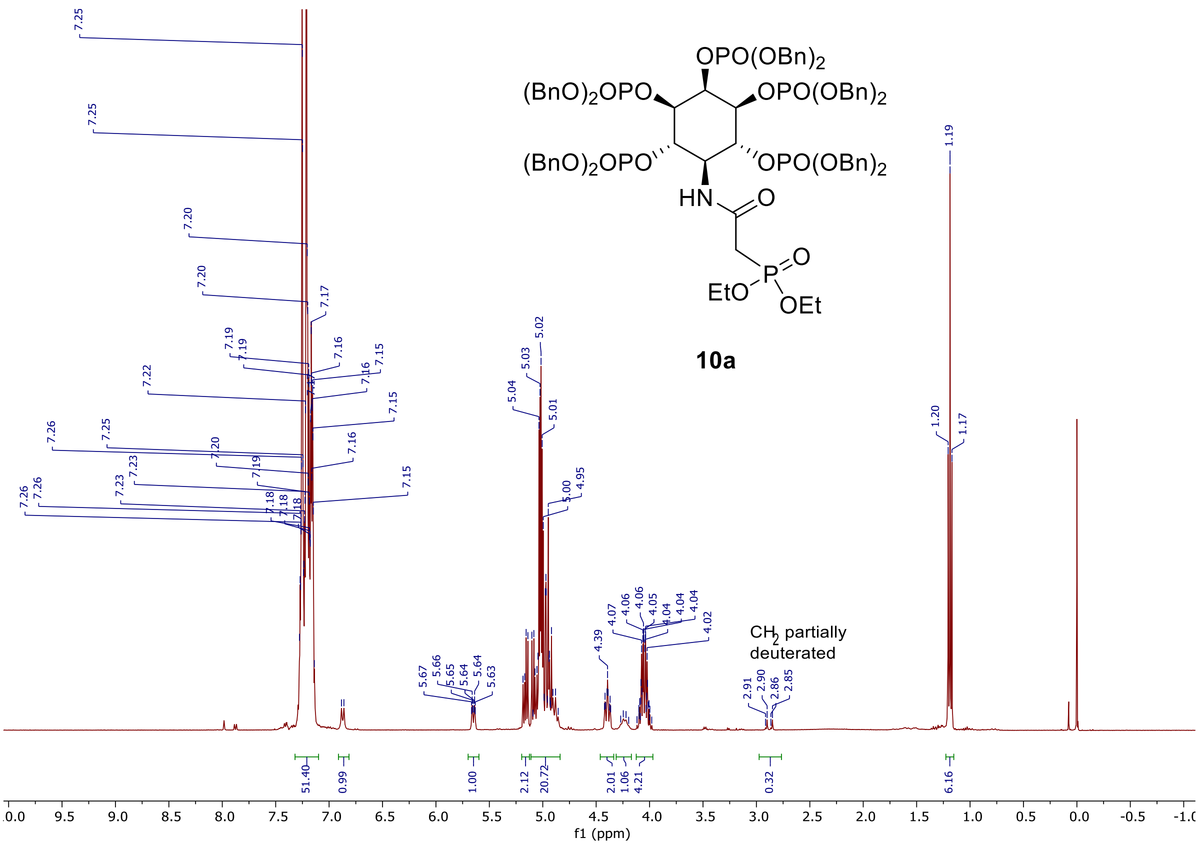

Compound **10a**; ^{13}C NMR (100 MHz, $CDCl_3$)

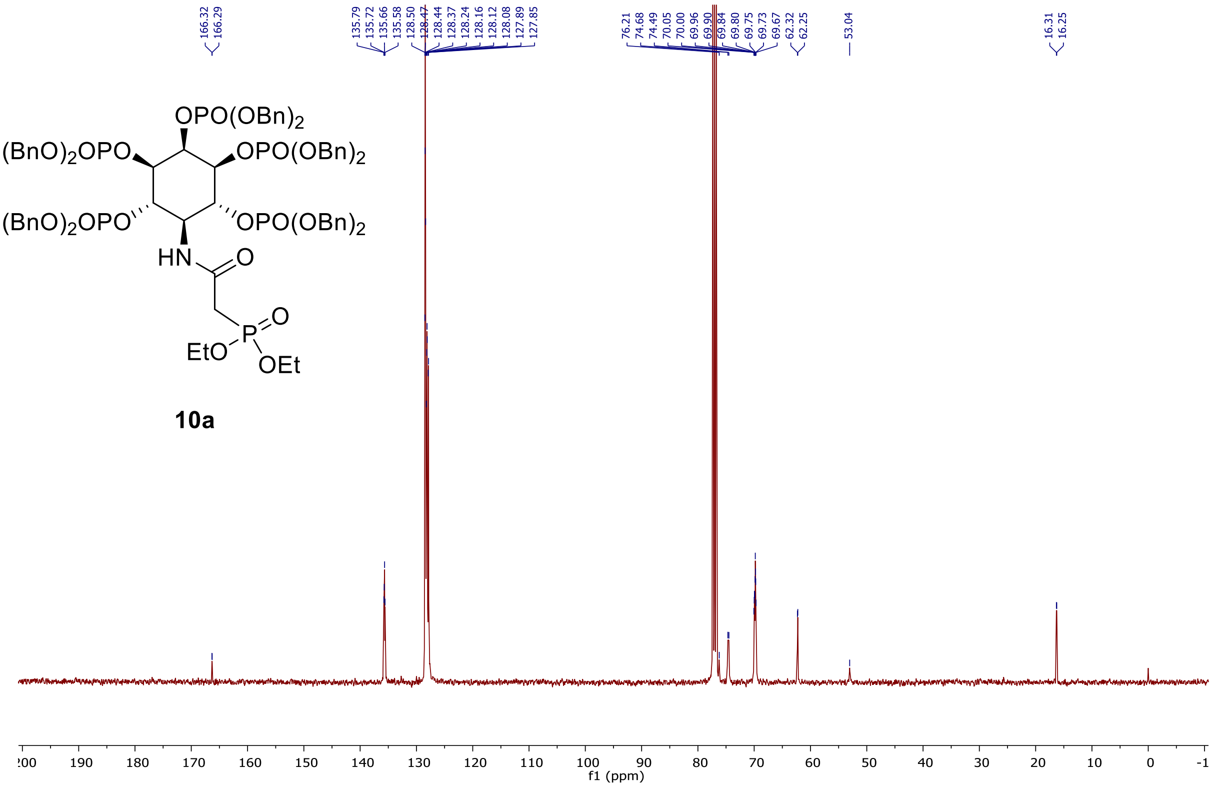

Compound **10a**; ^{31}P NMR (162 MHz, $CDCl_3$, ^{1}H-decoupled)

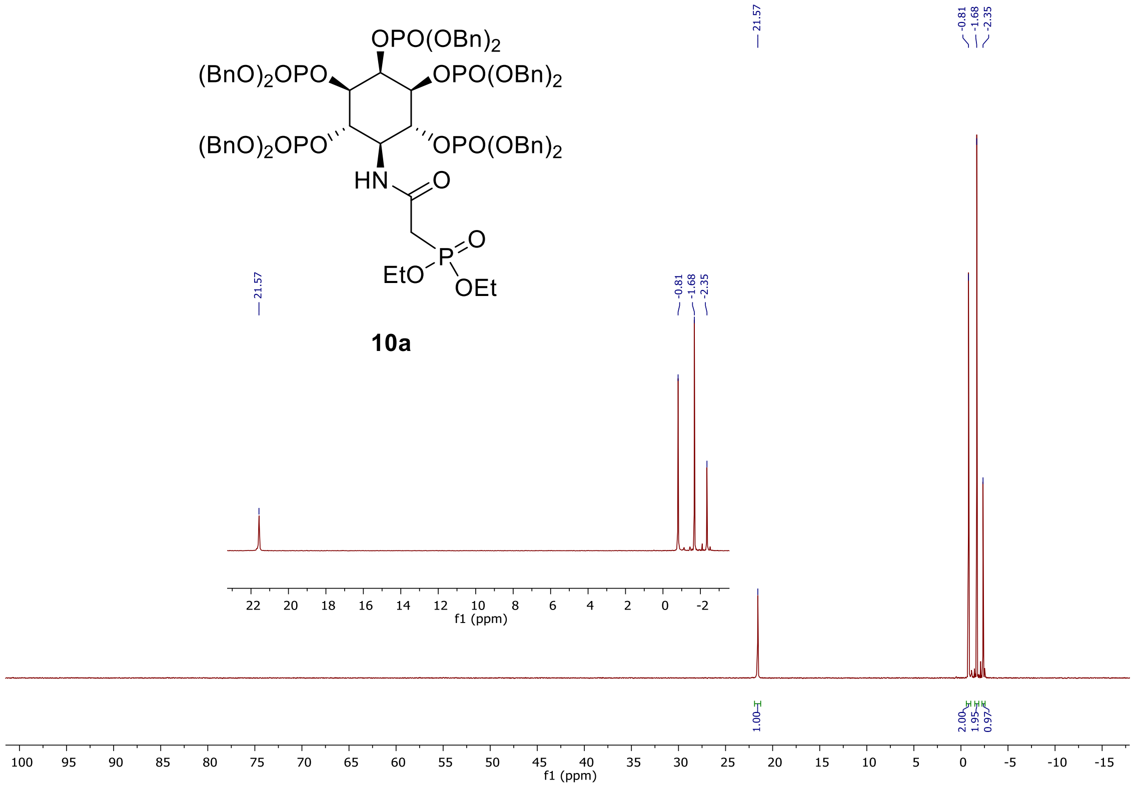

Supplement: Supplementary file 1 [file MD-010-C9MD00163H-s001.pdf]
